# Biocompatible exosomes derived from *Pinctada martensii* mucus for therapeutic melanin regulation via α-MSH/NF-κB/MITF pathway

**DOI:** 10.1093/rb/rbaf072

**Published:** 2025-07-03

**Authors:** Dandan Mo, Weihao Zheng, Zixin Gao, Ke Ma, Ke Yang, Tao Zeng, Chaozheng Qin, Yan Luo, Li Zheng, Sheng Xu

**Affiliations:** Collaborative Innovation Centre of Regenerative Medicine and Medical Bioresource Development and Application Co-Constructed by the Province and Ministry, Guangxi Key Laboratory of Regenerative Medicine, Guangxi Engineering Center in Biomedical Materials for Tissue and Organ Regeneration, The First Affiliated Hospital of Guangxi Medical University, Nanning 530021, China; Pharmaceutical College, Guangxi Medical University, Nanning 530021, China; Collaborative Innovation Centre of Regenerative Medicine and Medical Bioresource Development and Application Co-Constructed by the Province and Ministry, Guangxi Key Laboratory of Regenerative Medicine, Guangxi Engineering Center in Biomedical Materials for Tissue and Organ Regeneration, The First Affiliated Hospital of Guangxi Medical University, Nanning 530021, China; Pharmaceutical College, Guangxi Medical University, Nanning 530021, China; Life Sciences Institute, Guangxi Medical University, Nanning 530021, China; Collaborative Innovation Centre of Regenerative Medicine and Medical Bioresource Development and Application Co-Constructed by the Province and Ministry, Guangxi Key Laboratory of Regenerative Medicine, Guangxi Engineering Center in Biomedical Materials for Tissue and Organ Regeneration, The First Affiliated Hospital of Guangxi Medical University, Nanning 530021, China; Pharmaceutical College, Guangxi Medical University, Nanning 530021, China; Department of Plastic & Cosmetic Surgery, The First Affiliated Hospital of Guangxi Medical University, Nanning 530021, China; Department of Plastic & Cosmetic Surgery, The First Affiliated Hospital of Guangxi Medical University, Nanning 530021, China; Collaborative Innovation Centre of Regenerative Medicine and Medical Bioresource Development and Application Co-Constructed by the Province and Ministry, Guangxi Key Laboratory of Regenerative Medicine, Guangxi Engineering Center in Biomedical Materials for Tissue and Organ Regeneration, The First Affiliated Hospital of Guangxi Medical University, Nanning 530021, China; Collaborative Innovation Centre of Regenerative Medicine and Medical Bioresource Development and Application Co-Constructed by the Province and Ministry, Guangxi Key Laboratory of Regenerative Medicine, Guangxi Engineering Center in Biomedical Materials for Tissue and Organ Regeneration, The First Affiliated Hospital of Guangxi Medical University, Nanning 530021, China; Life Sciences Institute, Guangxi Medical University, Nanning 530021, China; Collaborative Innovation Centre of Regenerative Medicine and Medical Bioresource Development and Application Co-Constructed by the Province and Ministry, Guangxi Key Laboratory of Regenerative Medicine, Guangxi Engineering Center in Biomedical Materials for Tissue and Organ Regeneration, The First Affiliated Hospital of Guangxi Medical University, Nanning 530021, China; Collaborative Innovation Centre of Regenerative Medicine and Medical Bioresource Development and Application Co-Constructed by the Province and Ministry, Guangxi Key Laboratory of Regenerative Medicine, Guangxi Engineering Center in Biomedical Materials for Tissue and Organ Regeneration, The First Affiliated Hospital of Guangxi Medical University, Nanning 530021, China; Pharmaceutical College, Guangxi Medical University, Nanning 530021, China; Life Sciences Institute, Guangxi Medical University, Nanning 530021, China; Collaborative Innovation Centre of Regenerative Medicine and Medical Bioresource Development and Application Co-Constructed by the Province and Ministry, Guangxi Key Laboratory of Regenerative Medicine, Guangxi Engineering Center in Biomedical Materials for Tissue and Organ Regeneration, The First Affiliated Hospital of Guangxi Medical University, Nanning 530021, China; Life Sciences Institute, Guangxi Medical University, Nanning 530021, China

**Keywords:** exosomes of *Pinctada martensii* mucus, melanin production, tyrosinase activity, NF-κB signaling pathway

## Abstract

Abnormal melanin production can lead to various pigmentary disorders, which significantly affect patients’ quality of life and overall health. However, current clinical melanogenesis inhibitors have adverse side effects such as skin dryness, itching, erythema, etc. In this study, we used naturally isolated exosomes derived from *Pinctada martensii* mucus (PMMEXOs) and investigated the effects on melanin synthesis based on B16-F10 melanoma cells and zebrafish. We demonstrated that PMMEXOs effectively inhibited melanin production while exhibiting excellent biocompatibility. To elucidate the underlying mechanisms, RNA sequencing and bioinformatics analysis were employed, identifying 556 differentially expressed genes associated with PMMEXOs treatment. Kyoto Encyclopedia of Genes and Genomes (KEGG) pathway analysis revealed the involvement of the NF-κB signaling pathway in the regulation of melanogenesis. Further mechanistic studies confirmed that PMMEXOs significantly reduced tyrosinase activity and melanin content, accompanied by the downregulation of critical melanogenesis-related genes and proteins, including MITF, TYR, TYRP-1 and TRP-2. Notably, the anti-melanogenic effects of PMMEXOs were mediated by activation of the NF-κB signaling pathway, underscoring their regulatory role in melanin biosynthesis. Additionally, microRNA (miRNA) sequencing of PMMEXOs identified specific miRNAs implicated in immune regulation and modulation of the NF-κB pathway, further supporting their mechanistic involvement in melanin inhibition. These findings collectively position PMMEXOs as a promising and innovative therapeutic strategy for the prevention and treatment of pigmentary disorders such as melasma, age spots and wrinkles.

## Graphical abstract

**Figure:**
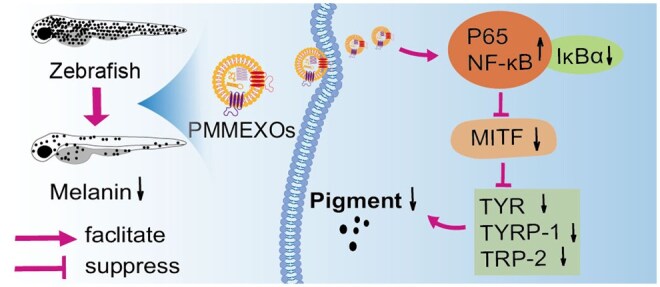

## Introduction

Dysregulation in melanin biosynthesis is associated with dermatological conditions, including post-inflammatory hyperpigmentation, melasma, lentigo and melanoma [1, 2]. These disorders, arising from aberrant melanin production, impose an escalating economic burden globally [3], as incidence rates and treatment costs rise annually [4, 5]. Effective management of hyperpigmentation disorders and skin cancer prevention, thus, necessitates the inhibition of melanin synthesis [6]. Therapeutic agents targeting melanin production include hydroquinone, kojic acid, arbutin [7] and various compounds derived from botanical extracts [8, 9]. Despite their efficacy in mitigating excess melanin, these agents have side effects, such as xerosis, pruritus, erythema, contact dermatitis and hypopigmentation [10, 11]. These adverse effects limit their widespread clinical application. Consequently, there is an urgent requirement for efficient treatment approaches that exhibit high safety profiles and clear mechanisms of action in inhibiting melanin synthesis.

Exosomes (EXOs), encapsulated by a phospholipid bilayer, are natural carriers of bioactive molecules like proteins, RNA and lipids [12, 13]. Their superior biocompatibility, low immunogenicity and targeted delivery capabilities make them promising as biomarkers, therapeutic agents and drug delivery vehicles [14–16]. Studies show mesenchymal stem cell (MSC)-derived exosomes promote bone regeneration, while exosomes from dermal papilla cells support wound healing [17, 18]. Natural sources such as milk, pork and lemon also demonstrate therapeutic potential [19–21]. However, challenges like safety, scalability and production costs highlight the need for alternative, efficient exosome sources.

Recent advancements have been made in the development of exosomes from marine sources, such as oysters, cod and sargassum [22, 23]. These marine-derived exosomes have shown promise in various therapeutic applications, notably in treating osteoporosis and inhibiting melanin synthesis in MNT-1 human melanoma cells via the α-MSH pathway. This provides a substantive basis for advancing therapeutically effective exosome development. *Pinctada martensii* (PM), also referred to as the Hepu pearl oyster, produces not only cultured pearls but also significant quantities of oyster meat, approximately 2000–3000 tons annually [24]. The mucus associated with this oyster meat, typically discarded as waste, offers substantial potential for resource development. Investigations into the biological activities of PM mucus have unveiled attributes such as antioxidant, anti-photoaging and wound-healing properties [25–27]. EXOs, capable of carrying various bioactive molecules, including peptides [28–30], miRNA [31, 32] and cytokines [33, 34], exhibit significant biological functions in skin protection. Notably, the W3 peptide found in PM meat has been shown to markedly reduce tyrosinase activity in B16-F10 cells, thus, enhancing cellular antioxidant capabilities [35]. Moreover, exosomes derived from *Pinctada martensii* mucus (PMMEXOs) have shown the capacity to reduce inflammation in Human Keratinocytes Cells (HaCaT) cells through the NF-κB/NLRP3/MAPK signaling pathway and exhibit potential in hindering osteosarcoma growth [36, 37]. Despite their multifaceted biological activities, the functions of these exosomes are still under exploration. Prior research has demonstrated that activating the NF-κB signaling pathway can reduce tyrosinase promoter activity, thereby decreasing tyrosinase gene expression and melanin synthesis [38]. Given that PMM-derived exosomes can activate the NF-κB pathway, it is hypothesized that PMMEXOs may also possess melanin-inhibiting bioactivity. However, the detailed impacts of PM-derived exosomes on cellular melanin production and the related mechanisms are still not fully understood.

**Figure 1.**
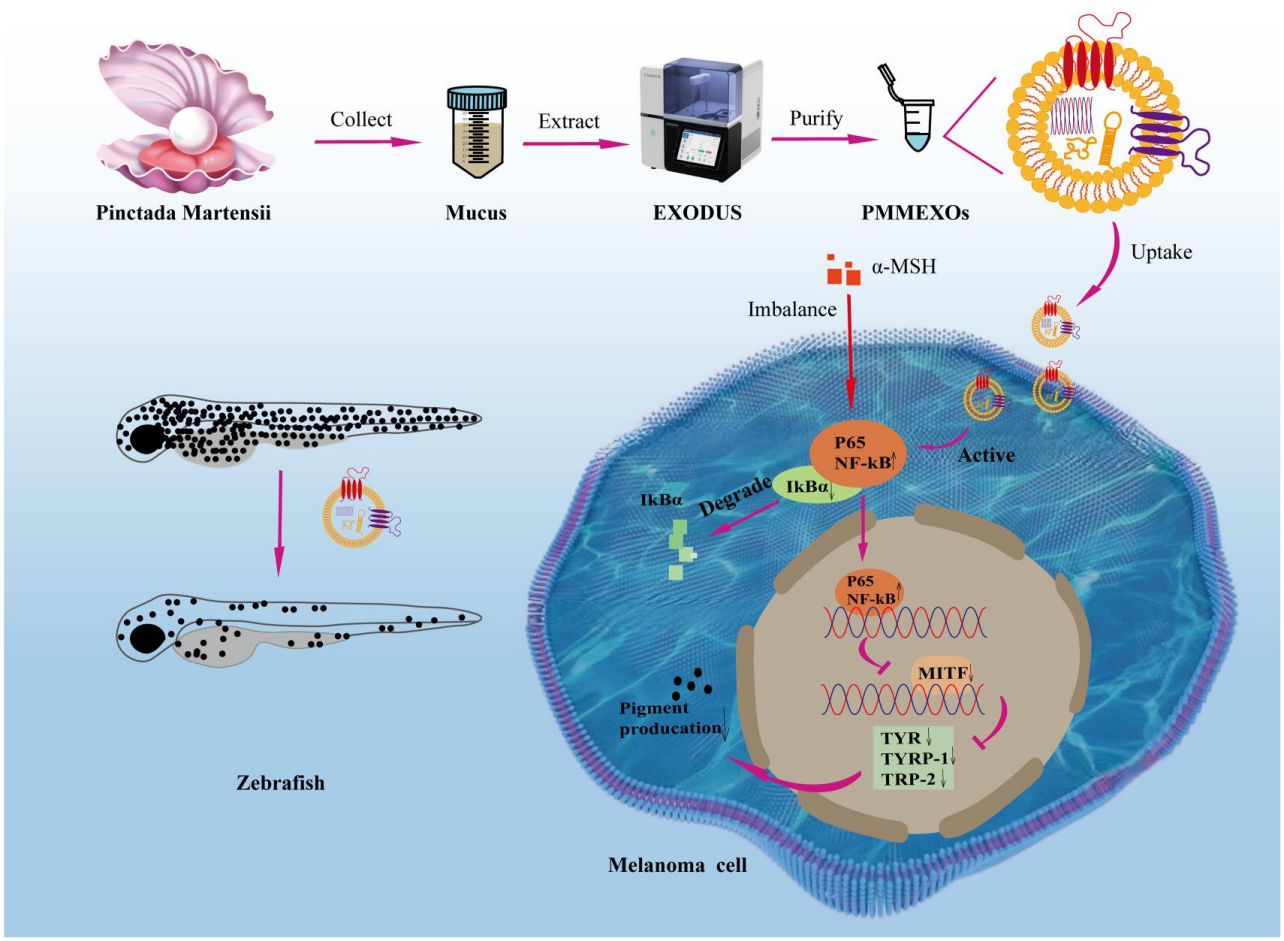
Exosomes derived from *Pinctada martensii* mucus (PMMEXOs) inhibit melanin production by activating the NF-κB signaling pathway.

In our research, we aim to rigorously explore the melanin-reducing influence of PMMEXOs and their underlying mechanistic pathways (Figure 1). We isolated and characterized PMMEXOs to analyze their bioactive components. Subsequently, we assessed the impact of PMMEXOs on melanin synthesis and associated enzyme functions *in vitro* using melanoma cell models, further elucidating their mechanisms via transcriptomic analysis to assess differential gene expression. Finally, the melanin inhibition potential of PMMEXOs was evaluated in zebrafish models. Our findings reveal that PMMEXOs inhibit melanin production by boosting the NF-κB signaling pathway, offering new perspectives for treating pigmentary disorders like melasma and post-inflammatory hyperpigmentation.

## Materials and methods

### Materials and reagents

*Pinctada Martensii* was sourced from Beishan Pearl Culture Co., Ltd., Beihai City, Guangxi. Mouse melanoma cells (B16-F10) were purchased from Procell Biotech, Wuhan, China. Obtained FBS, DMEM, penicillin–streptomycin and 0.25% trypsin-EDTA from Gibco, New York, NY, USA. Exosome Red Fluorescent Label staining (PKH26) was acquired from Umibio Biotechnology Co., Ltd., Shanghai, China. 4', 6-diamino-2-phenylindole (DAPI), immunofluorescence (IF) staining kit-Anti-rabbit Alexa Fluor 488, immunostaining permeable solution (Triton X-100), RIPA cracking buffer were all obtained from Beyotime Biotechnology, China. α-Melanocyte stimulating hormone (α-MSH), L-dihydroxyphenylalanine (L-DOPA), α-Arbutin (α-Ar) are all from Shanghai Maclin Biochemical Technology Co., Ltd. The TNF-α protein, BAY 11-7082, was purchased from MedChemExpress (MCE). The TRIzol kit, HiScript III RT SuperMix (with gDNA wiper) and ChamQ Universal SYBR qPCR Master Mix were secured from Vazyme, Nanjing, China. MITF antibody (13092-1-AP), Tyrosinase antibody (21995-1-AP), TYRP-1 antibody (16672-1-AP), TRP-2 antibody (68563-1-Ig), NF-κB p65 (66535-1-Ig), IκBα (664181-IG), β-actin (81115-1-RR) and HRP labeled rabbit/mouse secondary antibodies were purchased from Proteintech Group.

### Extraction of PMMEXOs

*Pinctada martensii* mucus (PMM) was centrifuged at 500 × *g* centrifuge for 10 min at 4°C to eliminate dead cells, then centrifuged again at 2000 × *g* for 10 min to collect the supernatant. Dilution and filtration of the supernatant precede its use in the EXODUS system for EXOs purification [39]. Detailed EXODUS protocol for isolating EXOs from the PMM [39, 40]: PMM samples (10 mL) were collected in 15 mL test tubes and centrifuged at 2000 × *g* for 10 min at 4°C. The supernatant was then transferred to a new test tube and filtered through a 0.22 μm needle filter, with the filtrate collected in a separate 15 mL tube. The EXODUS system was then initiated, and the prepared sample tubes were loaded onto the sample rack. The isolation parameters were set as follows: negative pressure: −30 kPa, conversion time: 10 s, and sample cleaning cycles: 2. The “Out” button was pressed to load the sample into the EXODUS device, and the “Start Analysis” button was pressed to begin exosome isolation. Once the analysis was completed, the EXODUS device automatically ejected the sample. The exosome solution was collected using a pipette and resuspended to 200 μL in a 1.5 mL centrifuge tube containing 1 × PBS. The isolated exosomes were resuspended in PBS, their protein concentration measured, and the samples stored at −80°C.

### Transmission electron microscope

PMMEXOs were retrieved from −80°C storage, equilibrated on ice and centrifuged. A 10 μL aliquot was applied to a copper grid, air-dried after staining with 3% phosphotungstic acid and examined under a transmission electron microscope (TEM).

### Particle size and concentration analysis

A 0.5 mL aliquot of PMMEXOs was diluted with PBS to attain a total volume of 2.0 mL, then, carefully placed in a spectroscopic well plate to prevent bubble entrapment, and analyzed for particle size. Subsequently, 0.8 mL of the diluted PMMEXOs was slowly pipetted into a disposable capillary tube for Zeta potential measurement, ensuring the tube was properly seated in the spectroscopic cell. We further confirmed the purity and integrity of the isolated exosomes by using the Nanocoulter I particle size analyzer. EXOs particles at a concentration of 10 ng/mL were suspended in PBS and injected into the sample chamber of the Nanocoulter I. The EXOs particles were measured based on Brownian motion and their diffusion coefficient. Data analysis was performed using the corresponding software.

### Cellular uptake

Following the manufacturer’s guidelines, PMMEXOs were labeled with the red fluorescent dye PKH26 (Umibio, China) for a 10 min incubation period, then, subjected to ultracentrifugation (100 000 × *g*, 70 min) to pellet the labeled EXOs. The pellet was reconstituted with 200 μL of PBS, and the labeled PMMEXOs were co-incubated with B16-F10 cells in a confocal dish for 24 h. Subsequently, DAPI was used to stain the cell nuclei, and the uptake and distribution of PMMEXOs were analyzed using a fluorescence microscope (Echo Revolve, China).

### Cell culture and cell viability assay

B16-F10 melanoma cells were cultured within a DMEM medium enriched with 10% FBS and a 1% penicillin-streptomycin solution, maintained at 37°C with 5% CO_2_. They were plated in 96-well plates at 1 × 10^4^ cells/mL and treated with PMMEXOs (0–300 μg/mL) for 24–72 h, and α-Ar (0, 50, 150, 500, 1000, 2000 μM) for 72 h. After 24 h stimulation with 0.1 μM α-MSH, B16-F10 cells, HaCaT cells and HUVECs were treated with PMMEXOs and α-Ar for 48 h, respectively. Cell survival was evaluated using the Cell Counting Kit-8 (CCK-8, Beyotime, China) at 450 nm after a 2 h incubation. Data from six replicates were analyzed in GraphPad Prism 9, with survival rate was assessed based on the formula: (OD of experiment—OD of blank)/(OD of control—OD of blank) × 100%. Cell morphology and counts were examined under a microscope and quantified with ImageJ.

### Determination of intracellular and extracellular melanin content

B16-F10 melanoma cells were grown in 6-well plates and exposed to 0.1 μM α-MSH for 24 h to stimulate melanin production. They were then incubated with PMMEXOs (0, 50, 100, 200 μg/mL) and 1 mM α-Ar for 48 h. Melanin in the medium was evaluated by visual pigmentation and confirmed quantitatively at 405 nm. Intracellular melanin was measured after cell lysis in a 1 M NaOH and 10% DMSO buffer and a 56°C incubation, with absorbance also read at 405 nm [41].

### The intracellular tyrosinase activity was measured

After treatment, permeabilization of the cells was achieved using 1% Triton X-100 in PBS and frozen at −20°C for 2 h. They were then thawed at ambient temperature. To measure melanin formation, 90 µl of a 5 mM L-DOPA solution was added, and the mixture was incubated at 37°C for 30 min. Melanin levels, reflecting tyrosinase activity, were measured at 490 nm using a spectrophotometer [42].

### Cellular immunofluorescence

B16-F10 melanoma cells were placed into 12-well plates at a rate of 2 × 10^5^ cells per well, followed by incubation for 24 h. They were then treated with 0.1 μM α-MSH for 24 h, followed by 48 h with PMMEXOs (0, 50, 100, 200 μg/mL) and 1 mM α-Ar. IF staining involved fixing the cells with a 4% paraformaldehyde solution, permeabilizing with 0.5% Triton X-100 and blocking non-specific binding with 1% BSA in PBST. An overnight incubation at 4°C was performed with primary antibodies specific to anti-MITF (13092-1-AP, Proteintech, Wuhan, China), anti-TYR (21995-1-AP, Proteintech, Wuhan, China), anti-TYRP-1 (16672-1-AP, Proteintech, Wuhan, China), anti-TRP-2 (68563-1-Ig, Proteintech, Wuhan, China) and anti-NF-κB p65 (66535-1-Ig, Proteintech, Wuhan, China). All the primary antibodies were specifically diluted at a ratio of 1:200. Then FITC-labeled secondary antibodies were used for incubation for 1 h. DAPI staining of the nuclei was followed by visualization of fluorescence under an inverted microscope. ImageJ software was used to quantify protein expression levels from the fluorescence intensity.

### *In vivo* study

Adult zebrafish were obtained from Nanjing Yishu Lihua Biotechnology Co., Ltd. Embryos from natural spawns were placed in 96-well plates, three per well and treated with PMMEXOs (0, 50, 100, 200 μg/mL) and 1 mM α-Ar for 72 h post-fertilization. Pigmentation effects were observed under a stereomicroscope and analyzed with Image pro-plus software to measure PMMEXOs-induced pigment changes.

The animal study was conducted in accordance with the guidelines for the care and use of experimental animals and was approved by the Use and Care Committee of the Experimental Animal Center of Guangxi Medical University (No. 202503001). In this study, 6-week-old female C57BL/6 mice were obtained from the Experimental Animal Center of Guangxi Medical University. After one week of acclimatization, the mice were randomly assigned to three groups: the control group, the UVB irradiation group and the PMMEXOs treatment group, which received a high concentration of 200 μg/mL PMMEXOs. Each group consisted of three mice. The mice’s ears were irradiated every other day with a Philips ultraviolet lamp, with a peak wavelength of 306 nm and an irradiation intensity of 180 mJ/cm^2^, for 2 weeks. Following UVB exposure, PMMEXOs were applied daily to the ear skin of the mice for a period of 4 weeks.

### Determination of melanin content and tyrosinase activity of zebrafish

Zebrafish embryos at 24 h post-fertilization were exposed to PMMEXOs (0, 50, 100, 200 μg/mL) and 1 mM α-Ar for 72 h. After treatment, embryos were sonicated and melanin levels were measured at 405 nm. Tyrosinase activity levels were measured by adding 100 μL of protein extract to L-DOPA (5 mM) and monitoring dopachrome formation at 490 nm after a 37°C incubation. Results from replicated experiments showed relative changes in melanin and tyrosinase activity versus controls, quantifying PMMEXOs’ impact on melanogenesis [43].

### RNA sequencing analysis

B16-F10 cells were cultured at 2 × 10^5^ cells/well in 6-well plates and stimulated with 0.1 μM α-MSH for 24 h before a 48 h incubation with 200 μg/mL PMMEXOs. Post-treatment, cells were harvested, snap-frozen in liquid nitrogen and stored at −80°C. RNA was extracted using a total RNA extraction kit (Magen, Guangzhou, China) and sent to Hangzhou Lianchuan Biotechnology Co., Ltd for RNA sequencing. The data analysis was conducted with statistical significance set at *P* < 0.05 and a minimum fold change threshold of ≥2, to identify genes that were significantly differentially expressed in response to PMMEXOs treatment.

### Reverse transcription-polymerase chain reaction

B16-F10 melanoma cells (2 × 10^5^ cells/well) were cultured in 6-well plates for 24 h. RNA was isolated using a Total RNA extraction kit and measured with a NanoDropTM 2000. cDNA was generated using a synthesis kit by Takara Bio, Japan. The SYBER green assay was applied for real-time quantitative PCR to assess gene expression levels. Roche’s Super Mix Plus (ID 50837000) was instrumental in detecting the target gene’s mRNA using an reverse transcription-polymerase chain reaction (RT-PCR) system by Roche, located in Basel, Switzerland, with GAPDH as the control gene. Primer details are Supplementary Table S1.

### Western blot analysis

The B16-F10 cells were first seeded in a 6-well plate (1 × 10^6^ cells/well) and incubated at 37°C for 24 h after treatment with the respective drugs. Total proteins were extracted by lysis with RIPA buffer (Beyotime, China). Nuclear and cytoplasmic proteins were isolated using the Nuclear and Cytoplasmic Protein Extraction Kit (Beyotime, China) and the concentrations were determined by NanoDrop 2000/2000c. Subsequently, the proteins were mixed with 4 × loading buffer at a 1:4 volume ratio and denatured at 95°C for 15 min. Equal amounts of proteins (20–30 µg) were then separated by 10% SDS-PAGE gel and transferred onto 0.45 µm PVDF membrane via a semi-dry method. The blot was treated with QuickBlock™ Blocking Buffer (Beyotime, China) solution at room temperature for 15 min. Then, incubated with primary antibodies at 4°C overnight, and with secondary antibodies for 2 h at room temperature. The primary antibodies included: anti-NF-κB p65 (66535-1-Ig, Proteintech, Wuhan, China), anti-IκBα (664181-IG, Proteintech, Wuhan, China) and anti-β-actin (81115-1-RR, Proteintech, Wuhan, China). The secondary antibodies consisted of HRP-conjugated Affinipure Goat Anti-Rabbit IgG(H + L) (Proteintech, China) and HRP-conjugated Affinipure Goat Anti-Mouse IgG (H + L) (Proteintech, China). The BeyoECL Star kit (Beyotime, China) was responsible for the formation of bands, which were then exposed using the Gel imaging instrument (BIO-RAD, Chemidoc XRS, USA). The Image Lab software (BIO-RAD, USA) was employed to analyze the protein bands, and quantitative analysis was conducted using GraphPad Prism 9.

### Microrna-seq analysis for PMMEXOs

Upon collection, PMMEXOs were maintained at −80°C for subsequent analysis. The samples were forwarded to Hangzhou LianChuan Bio-Tech Co., Ltd for high-throughput sequencing to profile microRNAs (miRNA-Seq). The analysis was facilitated by the bespoke ACGT101-miR (v4.2) software, which processed the raw data by eliminating 3' adapters and non-informative sequences, yielding Clean Data. The sRNA sequences were curated to include only those within the 18–26nt range, which is characteristic of miRNAs in animal species. Following this, the sequences were mapped against the mRNA, RFam and Repbase databases, excluding any miRNA annotations, to refine the dataset. The refined reads were then matched against known precursors and the reference genome to ascertain the presence of miRNAs. The differential expression analysis of miRNAs led to the identification of their putative target genes using TargetScan (v5.0) and miRanda (v3.3a). To elucidate the biological roles of these miRNAs, an annotation of their target genes was conducted using the GO (Gene Ontology, https://www.geneontology.org/) and KEGG (https://www.kegg.jp/) databases. Using a *P* values of <0.5 and a minimum fold change threshold of ≥2, we determined the enriched pathways.

### Statistical analysis

All means were statistically compared using GraphPad Prism 8 (GraphPad, USA). One-way ANOVA was used to perform multiple comparisons. *P* ≤ 0.05 (* or #), *P* ≤ 0.01 (** or ##) and *P* ≤ 0.001 (*** or ###) and *P* ≤ 0.0001 (**** or ####) were considered statistically significant, while ‘ns’ was used to denote not significant.

## Results

### EXOS isolation, characterization and cellular uptake

**Figure 2.**
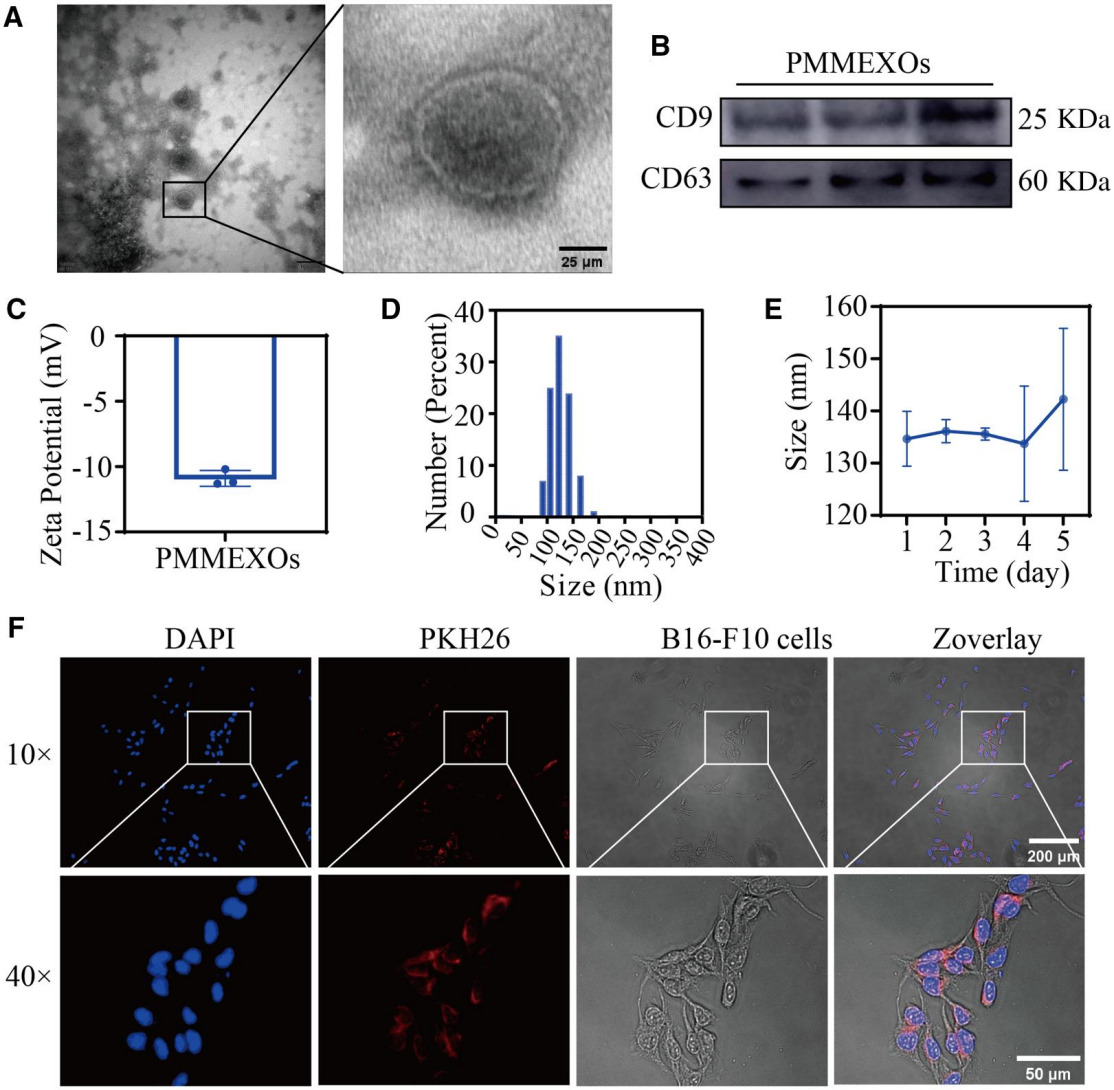
Characterization of PMMEXOs. (**A**) Morphology of PMMEXOs, observed by transmission electron microscopy (TEM). Scale bars = 25 μm. (**B**) Western blot analysis of EV biomarkers in PMMEXOs, including CD63 and CD9. (**C**) DLS represents the particle zeta of PMMEXOs. (**D**) DLS indicates the particle size of PMMEXOs. (**E**) Storage stability: PMMEXOs was stored at pH7.4 PBS, −80°C for 5 days and the particle size was determined. (**F**) The absorption of PMMEXOs by B16-F10 cells was observed by micrograph (magnification: 10 ×, scale bars = 200 μm) and magnification (magnification: 40 ×, scale bars = 50 μm). After incubation for 24 h, fluorescent PKH26-labeled PMMEXOs, nuclei stained with DAPI. *n* = 3.

EXOs were successfully isolated from the mucus of PM (Hepu pearl oyster) using specialized EXOs extraction equipment. The isolated EXOs were then assessed regarding their morphology, particle size, membrane potential and cellular uptake. TEM (Figure 2A) revealed that the exosomes (denoted as PMMEXOs) exhibited a characteristic cup-shaped morphology and intact membrane structure, consistent with the appearance of natural EXOs. NTA further demonstrated a spectrum of particle sizes extending from 30 to 150 nm, featuring a major peak at 134.7 nm. The stability of PMMEXOs was assessed over a five-day static storage period, showing no significant changes in particle size (Figure 2E), indicating their stability over time. **Western blot** (WB) analysis confirmed the presence of established exosome-associated biomarkers, CD9 and CD63, in the isolated PMMEXOs (Figure 2B). Additionally, the NTA revealed a zeta potential of −10.9 ± 1 mV and a general average diameter of 134 ± 10 nm, which was compatible with the morphology observed under TEM (Figure 2C and D). The initial concentration of EXOs was determined to be 4603.754 × 10^8^ particles/mL (Supplementary Figure S1A). The particle size distribution of the EXOs was relatively narrow, primarily ranging from 59 nm to 138 nm (Supplementary Figure S1B), which is consistent with the DLS results (Figure 2D). This distribution falls within the typical size range for EXOs (30-150 nm). These findings confirm that EXOs have been successfully isolated from the PMM.

To assess the uptake of PMMEXOs by B16-F10 melanoma cells, we labeled the EXOs with the fluorescent dye PKH26 and incubated them with the cells for 24 h. Fluorescence microscopy imaging revealed effective internalization of PMMEXOs by the melanoma cells, confirming that these EXOs can be readily taken up by the target cells (Figure 2F).

### Effect of PMMEXOs on melanin synthesis and tyrosinase activity in B16-F10 melanoma cells

**Figure 3.**
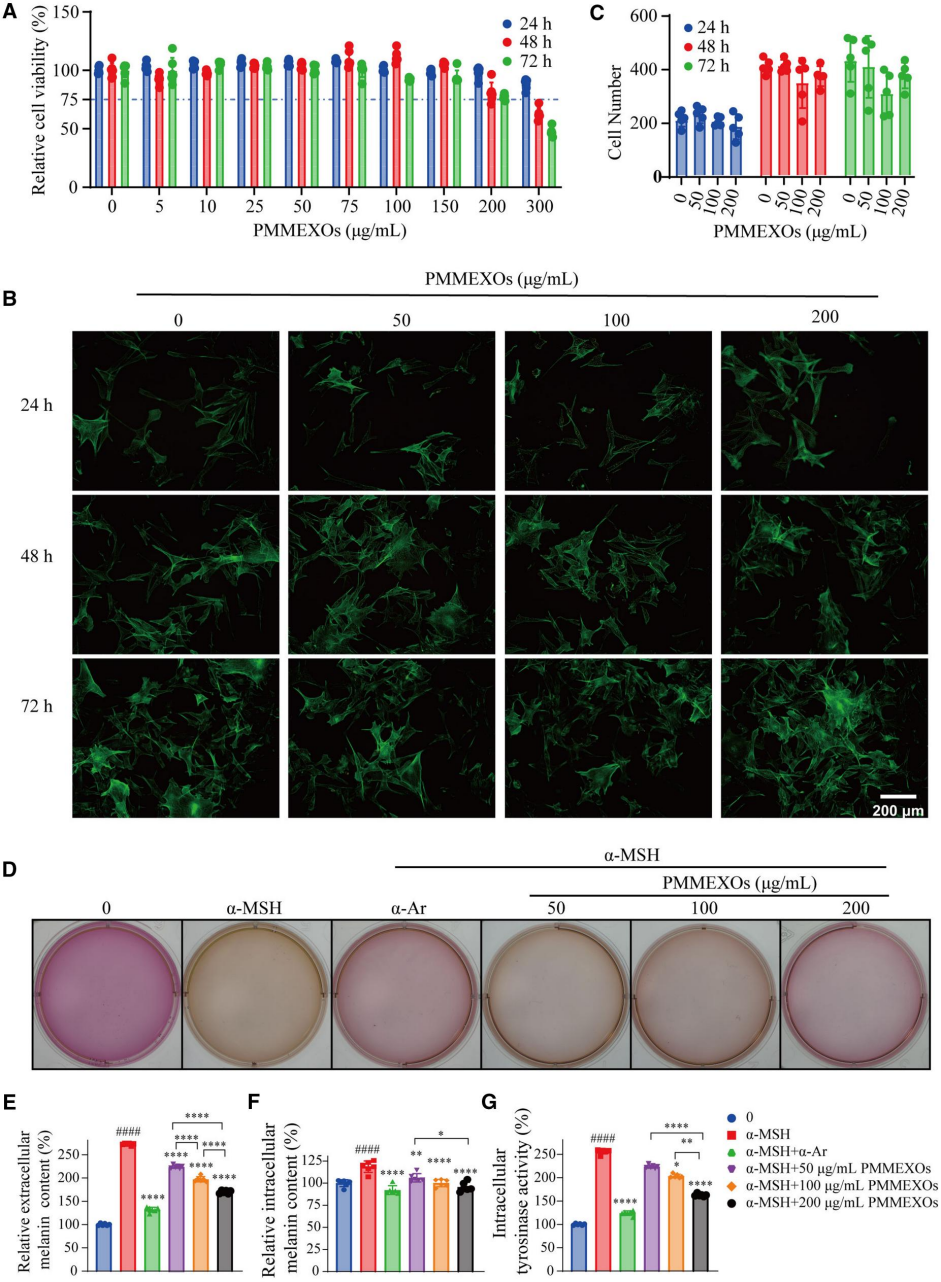
Effects of PMMEXOs on melanin secretion and tyrosinase activity in B16F10 melanoma cells. (**A**) PMMEXOs (0–300 μg/mL) treated B16F10 cells at 24 h, 48 h, 72 h relative cell viability, *n* = 6. (**B**) Microscopic images were collected every 24 h. Scale bars = 200 μm, *n* = 3. (**C**) Number of cells per 24 h, *n* = 5. (**D**) Change in medium colour after each well of drug treatment. Intracellular (**E**) and extracellular (**F**) measurements of melanin content, *n* = 6. (**G**) Evaluation of tyrosinase activity after PMMEXOs treatment, *n* = 6. Data were expressed as mean ± SD, and one-way analysis of variance was used for statistical significance. *P < 0.05, **P < 0.01, ***P < 0.001, ****P < 0.0001 compared with α-MSH model group, ####*P* < 0.0001 compared with the blank group.

To evaluate the biocompatibility of PMMEXOs, B16-F10 melanoma cells were exposed with varying concentrations of PMMEXOs (0–300 μg/mL), and cellular survival was assessed at different time points using the CCK-8 assay. The results indicated that cellular viability remained nearly unchanged (approximately 100%) when treated with PMMEXOs at concentrations ≤150 μg/mL. However, at concentrations >200 μg/mL, an observable decline in cellular viability was linked to the dose levels, with survival rates of 98.2%, 80.9% and 77.3% at 24, 48 and 72 h, respectively. Notably, treatment with 300 μg/mL of PMMEXOs significantly reduced cell viability, with survival rates of 62.6% at 48 h and 47.4% at 72 h (Figure 3A). Consequently, a concentration range of ≤200 μg/mL was selected for subsequent experiments, as this range did not adversely affect cell viability. The treated cells were stained using a live/dead cell staining kit, and fluorescence microscopy was used to capture the images, providing a clear visual representation of the cell viability, as shown in Supplementary Figure S2. When the concentration of PMMEXOs was below 200 μg/mL, cytotoxicity remained minimal, which aligns with the results from the CCK-8 assay (Figure 3A). Cell physiological functions are closely related to cell morphology [44]. To further investigate whether PMMEXOs influenced cell morphology, we examined the cytoskeletal structure following treatment. Cytoskeleton staining showed no discernible changes in the cell morphology after PMMEXOs treatment (Figure 3B), suggesting that PMMEXOs did not induce significant alterations in cellular structure. In addition, live cell counting via ImageJ demonstrated no significant alteration in the number of cells that are alive after 72 h of PMMEXOs treatment, which aligns with the findings from the CCK-8 assay (Figure 3C and Supplementary Figure S3). Together, these results indicate that PMMEXOs, at concentrations ≤200 μg/mL, do not affect cell growth and exhibit favorable biocompatibility.

To evaluate the cytotoxicity of α-Ar, B16-F10 cells were exposed to various concentrations of α-Ar, and cell viability was measured using the CCK-8 assay. The findings revealed that α-Ar maintained cell viability above 75% at concentrations up to 1000 μM, indicating no significant toxic effects on B16-F10 cells. However, at concentrations exceeding 1000 μM, α-Ar exhibited cytotoxicity (Supplementary Figure S4). Consequently, a concentration of 1 mM α-Ar was employed as the positive control in this study. To assess the biocompatibility of B16-F10 cells, as well as other normal skin cells such as HaCaT cells and HUVECs, the CCK-8 assay was utilized to evaluate cytotoxicity following induction with α-MSH. Neither the PMMEXOs group nor the α-Ar group exhibited toxic effects on B16-F10 cells, with cell survival rates exceeding 90% (Supplementary Figure S5A). The PMMEXOs group enhanced the proliferation of HaCaT cells, whereas the α-Ar group significantly inhibited cell growth, resulting in a cell survival rate of 59.8% (Supplementary Figure S5B). At a concentration of 50 μg/mL, PMMEXOs promoted the proliferation of HUVECs, while concentrations of 100 and 200 μg/mL had no cytotoxic effects on HUVECs. α-Ar showed no significant toxic effects on HUVECs, with a cell survival rate of 82.7% (Supplementary Figure S5C). In summary, PMMEXOs demonstrated superior cytocompatibility compared to α-Ar.

To investigate the effect of PMMEXOs on melanin production, we quantified both extracellular and intracellular melanin content in B16-F10 melanoma cells. The cells were stimulated with α-MSH and applied with varying concentrations of PMMEXOs (50, 100 and 200 μg/mL) for 48 h. Changes in the color of the culture medium, which darkened in response to melanin production, were observed. α-Ar was utilized to establish a positive baseline for melanin inhibition. As expected, α-MSH stimulation resulted in a significant increase in melanin synthesis. However, treatment with PMMEXOs led to a dose-dependent reduction in melanin production (Figure 3D). Additionally, we have included microscopic images that visually illustrate the distribution and content of melanin in the cells. In B16F10 cells, we observed that the inhibitory effect on melanin production improved with increasing concentrations of PMMEXOs (Supplementary Figure S6). Quantitative analysis of both extracellular and intracellular melanin content revealed that α-MSH stimulation increased extracellular and intracellular melanin by approximately 273% and 118%, respectively, compared to untreated controls. In contrast, both PMMEXOs and α-Ar significantly reduced the melanin content. PMMEXOs treatment led to a dose-dependent decrease in intracellular melanin content by 12%, 18% and 22% with levels of 50, 100 and 200 μg/mL, respectively (Figure 3E). Likewise, extracellular melanin content was reduced by 49%, 76% and 103% at the same concentrations (Figure 3F). These results underscore the melanin-inhibitory effects of PMMEXOs.

Furthermore, we assessed the impact of PMMEXOs on tyrosinase activity, a key enzyme involved in melanin synthesis. Treatment with PMMEXOs (200 μg/mL) markedly decreased tyrosinase levels in α-MSH-stimulated cells from 255.94% to 162.70%, in a dose-dependent style (Figure 3G). This indicates that PMMEXOs can modulate tyrosinase activity and thereby inhibit melanin production. Collectively, these results demonstrate that PMMEXOs exhibit significant anti-melanogenic properties, partly through the modulation of tyrosinase activity within melanoma cells. These findings lay the foundation to delve deeper of PMMEXOs as a therapeutic option for alleviating disorders of pigmentation, such as melasma and post-inflammatory hyperpigmentation and potentially for other diseases related to excessive melanin production.

### Quantitative analysis of melanin content and tyrosinase activity in B16-F10 cells

**Figure 4.**
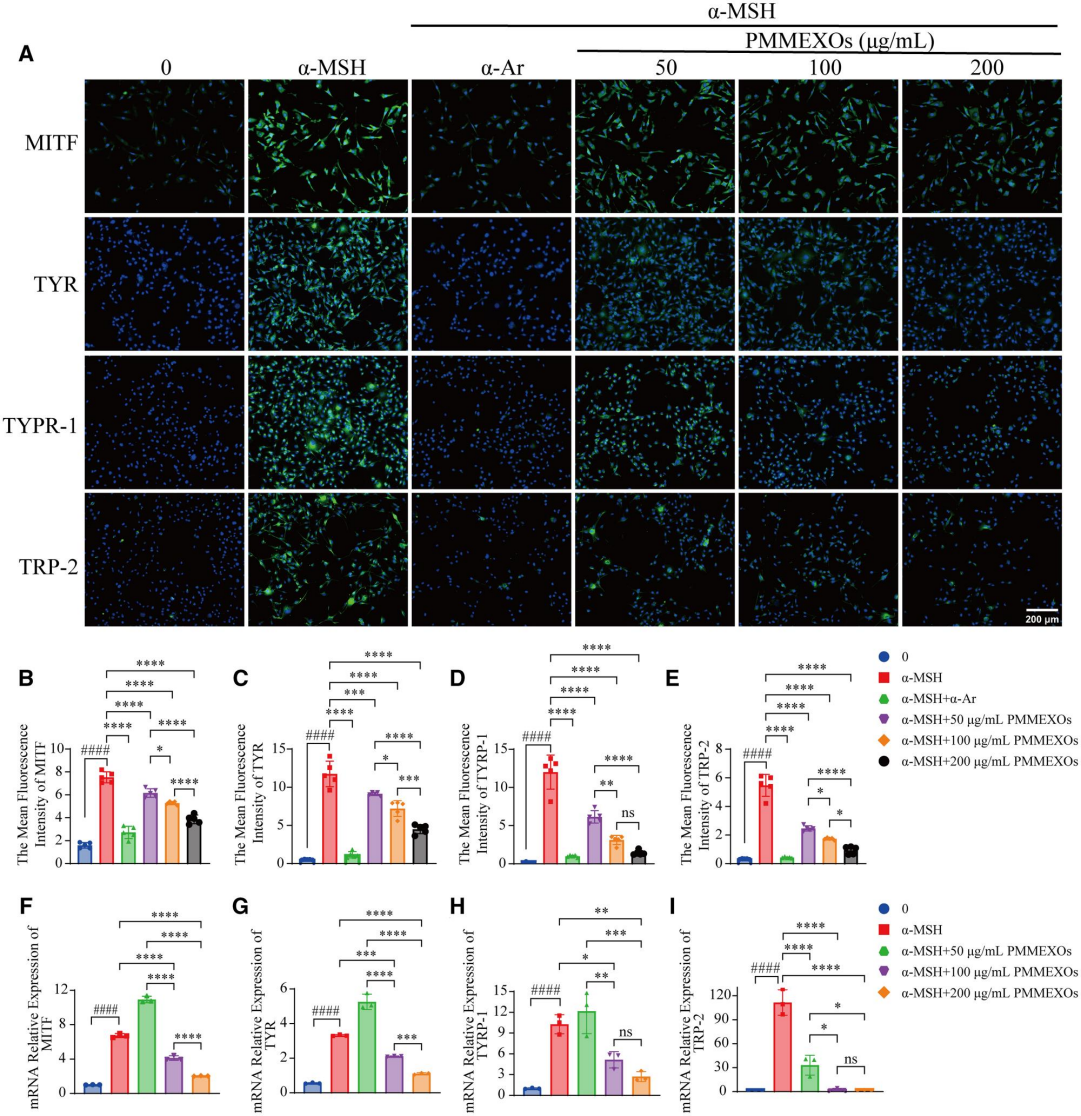
PMMEXOs inhibits the expression of melanin-related proteins and genes. (**A**) Immunofluorescence staining of MITF, TYR, TYRP-1 and TRP-2 proteins during melanin production. After incubation for 24 h, fluorescent of MITF, TYR, TYRP-1 and TRP-2 proteins, nuclei stained with DAPI. Scale bars = 200 μm. Quantitative fluorescence intensity analysis of MITF (**B**), TYR (**C**), TYRP-1 (**D**), TRP-2 (**E**) proteins. *n* = 5. The mRNA expression levels of MITF (**F**), TYR (**G**), TYRP-1 (**H**) and TRP-2 (**I**) in PMMEXOs treated cells were detected by RT-qPCR (*n* = 3). Data were expressed as mean ± SD, and one-way analysis of variance was used for statistical significance. n. s. no significant difference observed between groups. *P < 0.05, **P < 0.01, ***P < 0.001, ****P < 0.0001 compared with α-MSH model group, ####*P *< 0.0001 compared with the blank group.

Microphthalmia-associated transcription factor (MITF), a crucial regulator in transcription, controls tyrosinase activity and is essential for melanin synthesis, melanocyte differentiation, proliferation and survival [45]. MITF regulates melanin synthesis through the adjustment of multiple protein expression levels, including tyrosinase (TYR), tyrosinase-related proteins 1 (TRP-1) and 2 (TRP-2), and also influences cell cycle progression [46]. To investigate the impact of PMMEXOs regarding the synthesis of melanin and the function of tyrosinase, we assessed the expression levels of MITF, TYR, TRP-1 and TRP-2 using cellular IF and quantitative PCR (qPCR). Our IF results revealed that α-MSH treatment significantly enhanced the fluorescence signals for MITF, TYR, TRP-1 and TRP-2, indicating upregulation of these proteins in B16-F10 cells. In contrast, treatment with α-Ar or PMMEXOs markedly diminished the fluorescence intensities of these proteins (Figure 4A and Supplementary Figure S7). Notably, PMMEXOs reduced the expression levels of MITF, TYR, TRP-1 and TRP-2 in a dose-dependent process when compared against the α-MSH-induced group (Figure 4B–E). Further confirmation of these findings was obtained via qPCR, which demonstrated that α-MSH provocation boosted the mRNA expression of MITF, TYR, TRP-1 and TRP-2 in B16-F10 cells, correlating with the observed increases in melanin concentration and the performance of tyrosinase. However, PMMEXOs treatment at concentrations of 100 and 200 μg/mL dose-dependently reduced the mRNA expression levels of these genes (Figure 4F–I). These results indicate that PMMEXOs can effectively modulate the expression of key molecules participating in the synthesis of melanin and tyrosinase activity. We propose that PMMEXOs inhibit tyrosinase activity by regulating the expression of MITF, TYR, TRP-1 and TRP-2, ultimately suppressing melanin production.

### *In vivo* effects of PMMEXOs on melanin production

**Figure 5.**
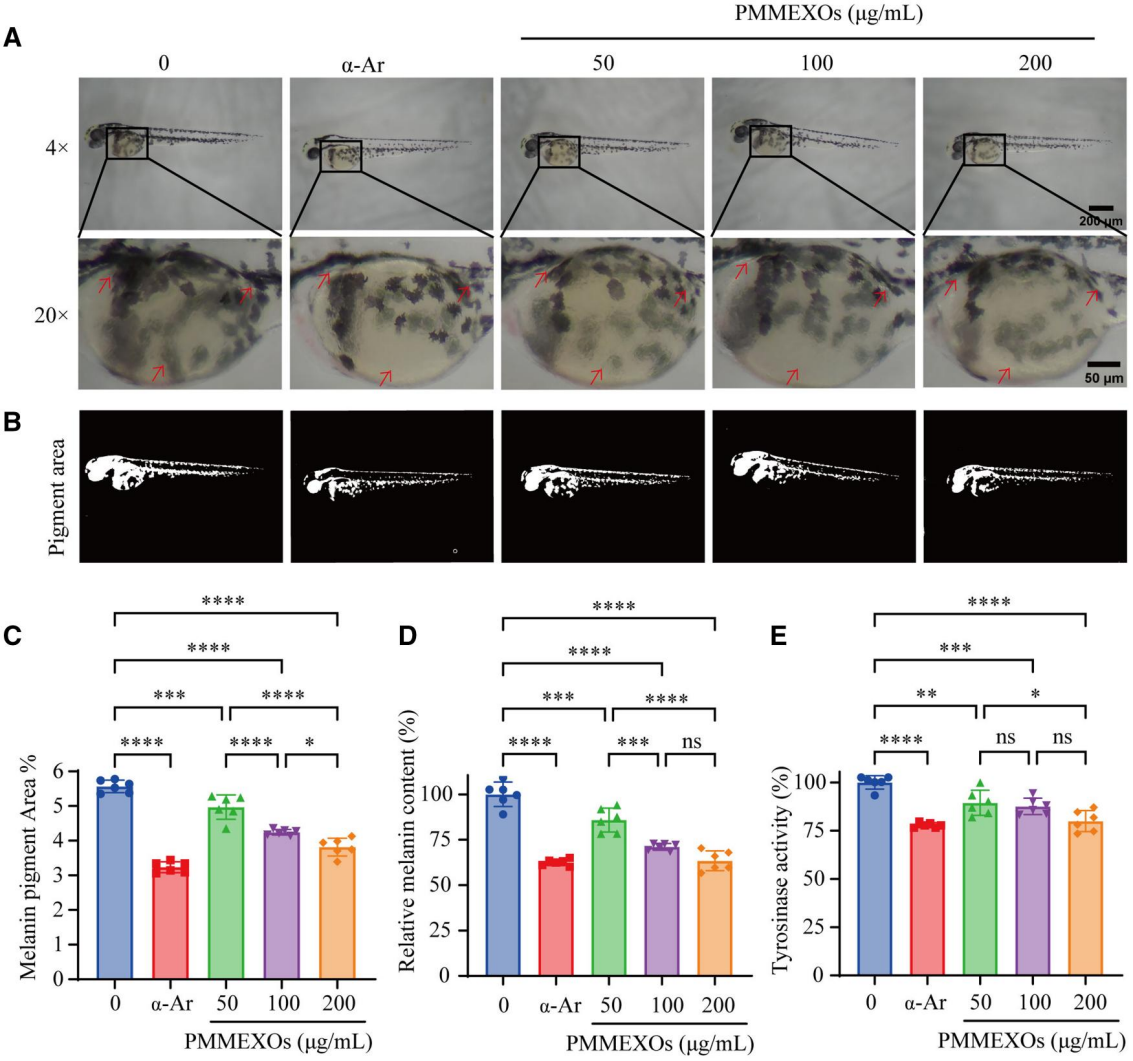
*In vivo* effects of PMMEXOs on inhibiting melanin production in zebrafish embryos. (**A**) Micrograph (magnification: 4×, scale bars = 200 μm) and magnification (magnification: 20×, scale bars = 50 μm) of juvenile zebrafish pigmentation. The arrows indicate melanin pigment. (**B**) ImageJ software for analysis of melanin deposits in zebrafish embryos. (**C**) Quantitative analysis of the relative melanin deposition area. (**D**) Relative melanin content in juvenile zebrafish. (**E**) tyrosinase activity in zebrafish juveniles. *n* = 6. Data were expressed as mean ± SD, and one-way analysis of variance was used for statistical significance. n. s. no significant difference observed between groups. *P < 0.05, **P < 0.01, ***P < 0.001, ****P < 0.0001 compared with α-MSH model group, ####*P *< 0.0001 compared with the blank group.

Zebrafish have been widely used as an *in vivo* model for studying melanin production due to their high genetic similarity to humans and the presence of melanocytes and melanosomes similar to those found in human skin. In addition, their small size, large sample volume and short experimental cycles make them an ideal model for studying melanin inhibition [47, 48]. To evaluate the *in vivo* anti-melanogenic potential of PMMEXOs, zebrafish larvae were treated with PMMEXOs, as well as α-Ar (a known melanin inhibitor), and melanin production was quantified. As expected, PMMEXOs treatment significantly reduced melanin deposition in the zebrafish larvae, with results comparable to α-Ar treatment (Figure 5A and B). In a concentration-dependent manner, PMMEXOs at concentrations of 50, 100 and 200 μg/mL inhibited melanin deposition. Notably, treatment with 200 μg/mL PMMEXOs resulted in a 31.5% reduction in melanin deposition on the surface of zebrafish, which was similar to the 41.8% reduction observed with α-Ar (Figure 5C). Biochemical analysis of melanin content in zebrafish embryos revealed a significant reduction in melanin levels upon PMMEXOs treatment. Specifically, treatment with 50, 100 and 200 μg/mL PMMEXOs led to an 85.82%, 71.10% and 63.40% reduction in melanin content, respectively, while α-Ar reduced melanin content by 62.56% (Figure 5D). Furthermore, as shown in Figure 5E, PMMEXOs treatment diminished tyrosinase activity in zebrafish embryos. In comparison to α-Ar, 200 μg/mL PMMEXOs also demonstrated a reduction in tyrosinase activity, reinforcing the inhibitory influence on melanogenesis.

**Figure 6.**
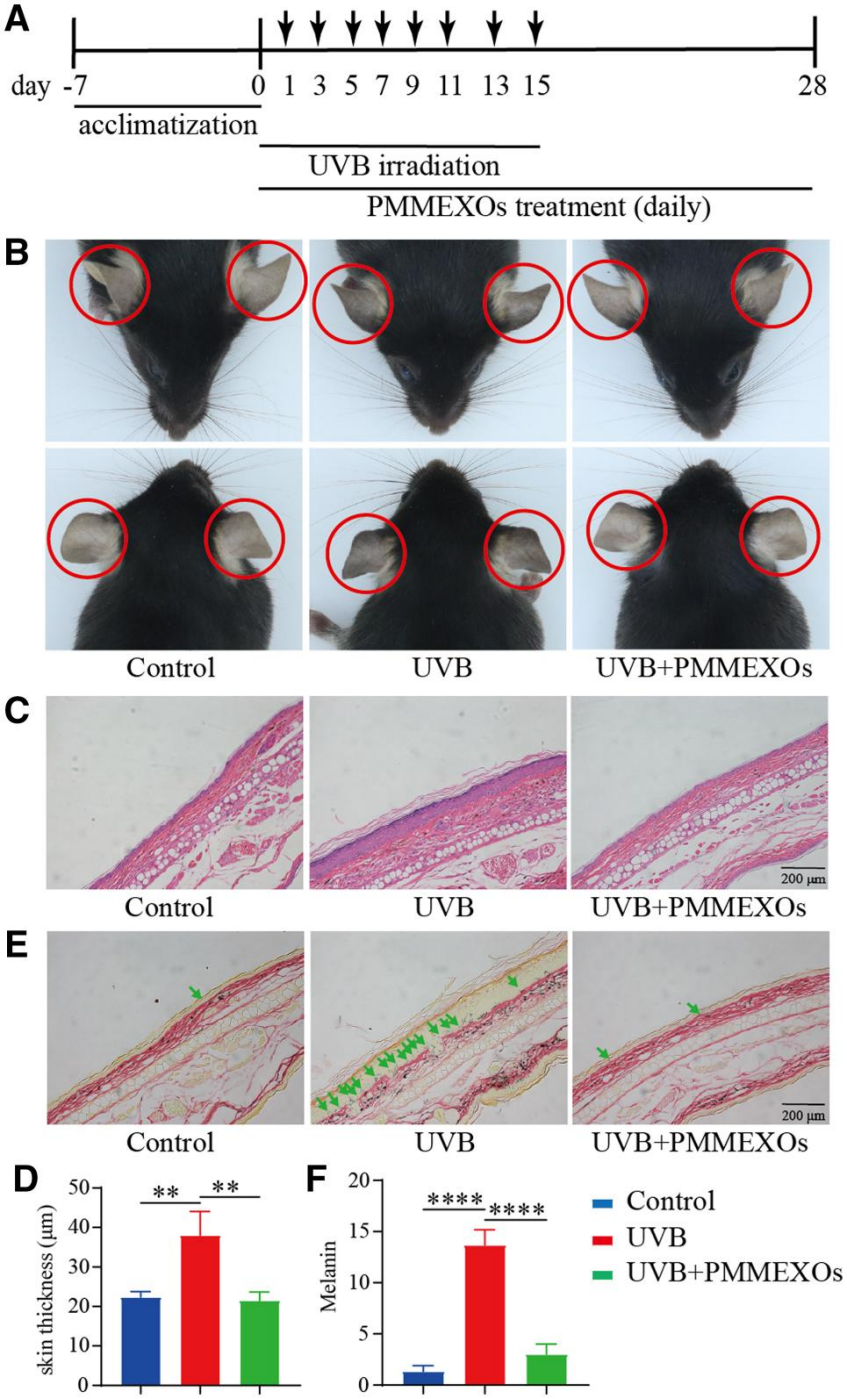
The effect of PMMEXOs on excessive pigmentation of the ear skin in C57BL/6 mice induced by UVB irradiation. (**A**) Time graph of the mouse experimental procedure. (**B**) Representative images of pigmentation on the skin of mouse ears. The circles indicate the areas irradiated by UVB. (**C**) Representative images of HE staining of mouse ear skin tissue. Scale bars =200 μm. (**D**) Statistical analysis of the corresponding epidermal thickness of the ear skin. (**E**) Representative images of Fontana–Masson staining of mouse ear skin tissue. The melanin pigment indicated by the arrow. Scale bars =200 μm. (**F**) Statistical analysis of the corresponding melanin content. *n* = 3, data are expressed as mean ± SD, one-way analysis of variance was used for statistical significance. **P < 0. 01, ****P < 0.0001.

After completing the experiment, photographs and skin samples were collected from the mice’s ear skin. The effect of PMMEXOs treatment on UVB-induced pigmentation was assessed through H&E and Fontana-Masson staining. UVB irradiation led to excessive pigmentation in the ear skin (the affected area is indicated by a red circle), while PMMEXOs treatment demonstrated a noticeable decolorization effect (Figure 6B). UVB exposure is known to induce skin hyperplasia. The H&E staining results revealed that UVB exposure triggered epidermal hyperplasia, whereas PMMEXOs treatment effectively reversed this effect (Figure 6C and D). These findings suggest that PMMEXOs can protect the skin from UVB-induced epidermal thickening and damage. Fontana-Masson staining was employed to detect melanin content. Under UVB irradiation, melanin synthesis was induced (highlighted by a green arrow), but PMMEXOs treatment led to a reduction in melanin content within the ear epidermis of the mice (Figure 6E and F).

These *in vivo* findings corroborate the anti-melanogenic properties observed *in vitro*.

### Global gene profile analysis of PMMEXOs treatment in B16-F10 cells

**Figure 7.**
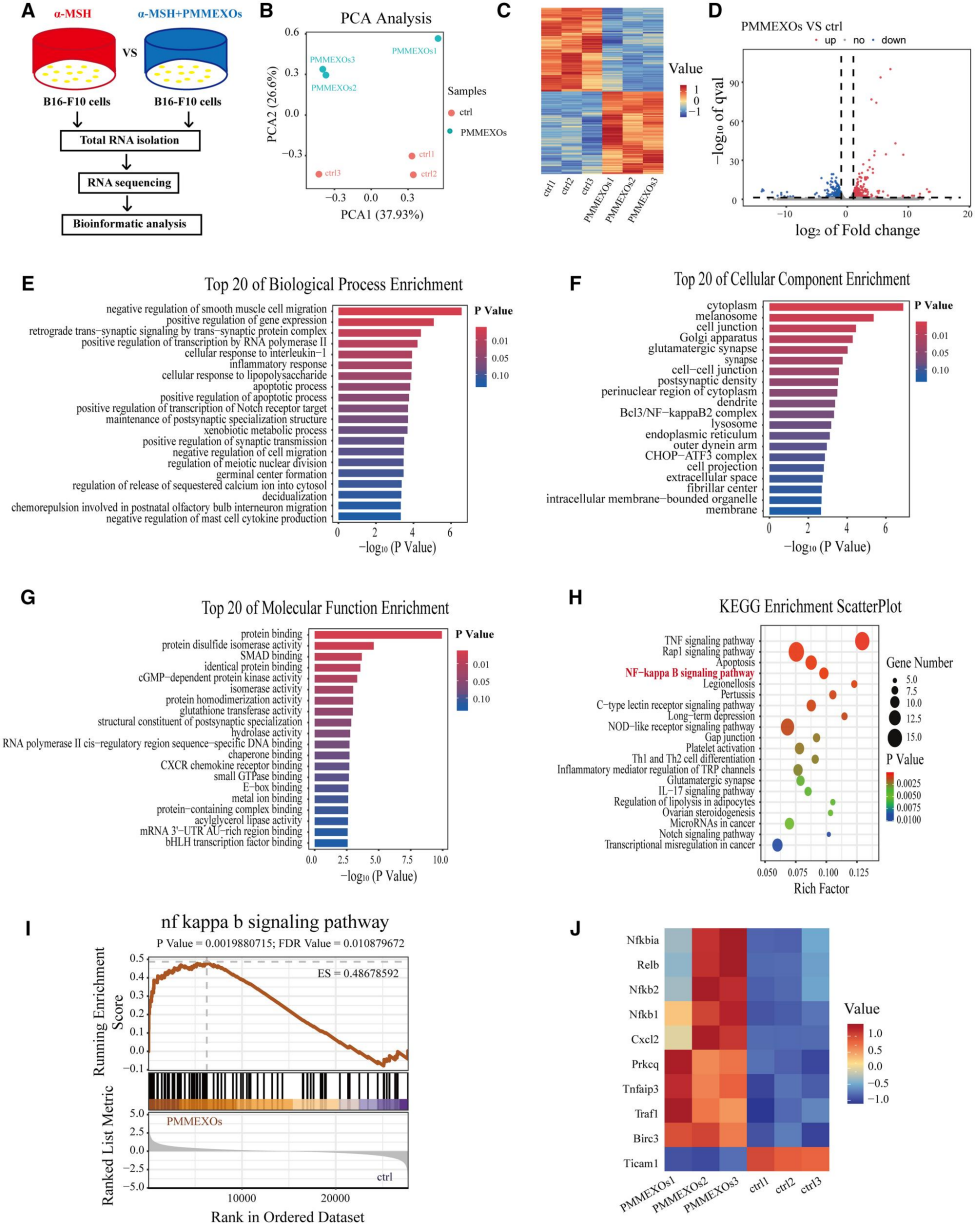
Analysis of RNA sequencing results and enrichment of differentially expressed genes. (**A**) Schematic of the RNA sequencing analysis of B16-F10 cells. (**B**) PCA analysis of two samples (**C**) Differential expression gene cluster heat maps for each sample. (**D**) Volcanic maps showing DEGs. The top 20 significantly enriched in GO terms, including biological processes (**E**), cell composition (**F**) and molecular function (**G**). (**H**) The top 20 significantly enriched KEGG pathways. (**I**) Enrichment map of NF-kappa B signaling pathway (nominal *P *< 0.05, FDR < 0.01, NES = 1.7455872). (**J**) Heat maps of gene aggregation in NF-κB signaling pathways.

Given that PMMEXOs at 200 μg/mL demonstrated a more pronounced effect on melanin production and tyrosinase activity, we sought to explore the broader transcriptional impact of PMMEXOs on gene expression in B16-F10 cells. Following transcriptome sequencing, a bioinformatics analysis was implemented on B16-F10 cells treated with 200 μg/mL PMMEXOs (PMMEXOs group) or without (control group) after α-MSH induction (Figure 7A). Principal component analysis (PCA) revealed clear separation between the control and PMMEXOs groups, with distinct clustering trends indicating significant changes in gene expression patterns upon PMMEXOs treatment (Figure 7B). A heatmap further illustrated the gene expression distribution across different samples, highlighting the global transcriptional changes induced by PMMEXOs (Figure 7C). Differentially expressed genes (DEGs) were established using a cutoff of |fold change| ≥1.5 and FDR < 0.05. A total of 556 genes were observed to be distinctively expressed, with 285 genes upregulated and 271 genes downregulated in the PMMEXOs-treated group (Figure 7D).

GO and KEGG pathway enrichment analyses were employed to explore the biological mechanisms of these DEGs. GO analysis demonstrated that the DEGs were primarily associated with biological processes (BP) such as ‘cellular response to interleukin-1’, ‘cellular response to lipopolysaccharide’ and ‘apoptotic process’ (Figure 7E). The DEGs were also enriched in cellular components (CC) such as ‘cytoplasm’, ‘melanosome (GO : 0042470)’ and ‘Bcl3/NF-kappaB2 complex’ (Figure 7F), as well as molecular functions (MF) like ‘SMAD binding’, ‘cGMP-dependent protein kinase activity’ and ‘CXCR chemokine receptor binding’ (Figure 7G). KEGG pathway analysis uncovered that the DEGs were substantially enriched in several pathways, including the TNF signaling pathway, Rap1 signaling pathway, apoptosis, NF-κB signaling pathway and NOD-like receptor signaling pathway (Figure 7H). Given the relevance of these pathways to melanin synthesis and immune response regulation, we specifically focused on the NF-κB signaling pathway. Gene set enrichment analysis (GSEA) revealed a positive correlation between PMMEXOs expression and the NF-κB signaling pathway (Figure 7I). Activation of the NF-κB pathway is known to influence melanocyte function and melanin production [49, 50]. Further analysis of genes involved in the NF-κB signaling pathway revealed significant differences in the expression of key regulators, including NF-κB family members (Nfkb1, Nfkb2, Relb), NF-κB signaling regulators (Traf1, Tnfaip3, Prkcq, Nfkbia), chemokines (Cxcl2) and apoptosis-related proteins (Birc3) (Figure 7J). These results suggest that PMMEXOs may restrain melanin synthesis by activating the NF-κB signaling pathway, thereby modulating the expression of downstream proteins such as MITF, TYR, TRP-1 and TRP-2, ultimately leading to the suppression of tyrosinase activity and melanin synthesis.

### RT-qPCR verified transcriptome sequencing data

The NF-κB complex, a critical regulator in immune responses, stress signaling, inflammation and apoptosis, consists of five proteins: NFKB1, NFKB2, RELA, RELB and REL [51]. Typically, the p50 and p65 subunits form a heterodimer, which remains inactive in the cytoplasm due to its interaction with IκB-α. Upon cellular stimulation by inflammatory signals, the IκB kinase (IKK) complex becomes activated, phosphorylating IκB-α. This phosphorylation marks IκB-α for ubiquitination and proteasomal degradation, releasing the NF-κB dimer, which then translocates to the nucleus to bind specific DNA sequences (κB sites) and initiate transcription of target genes [52, 53].

**Figure 8.**
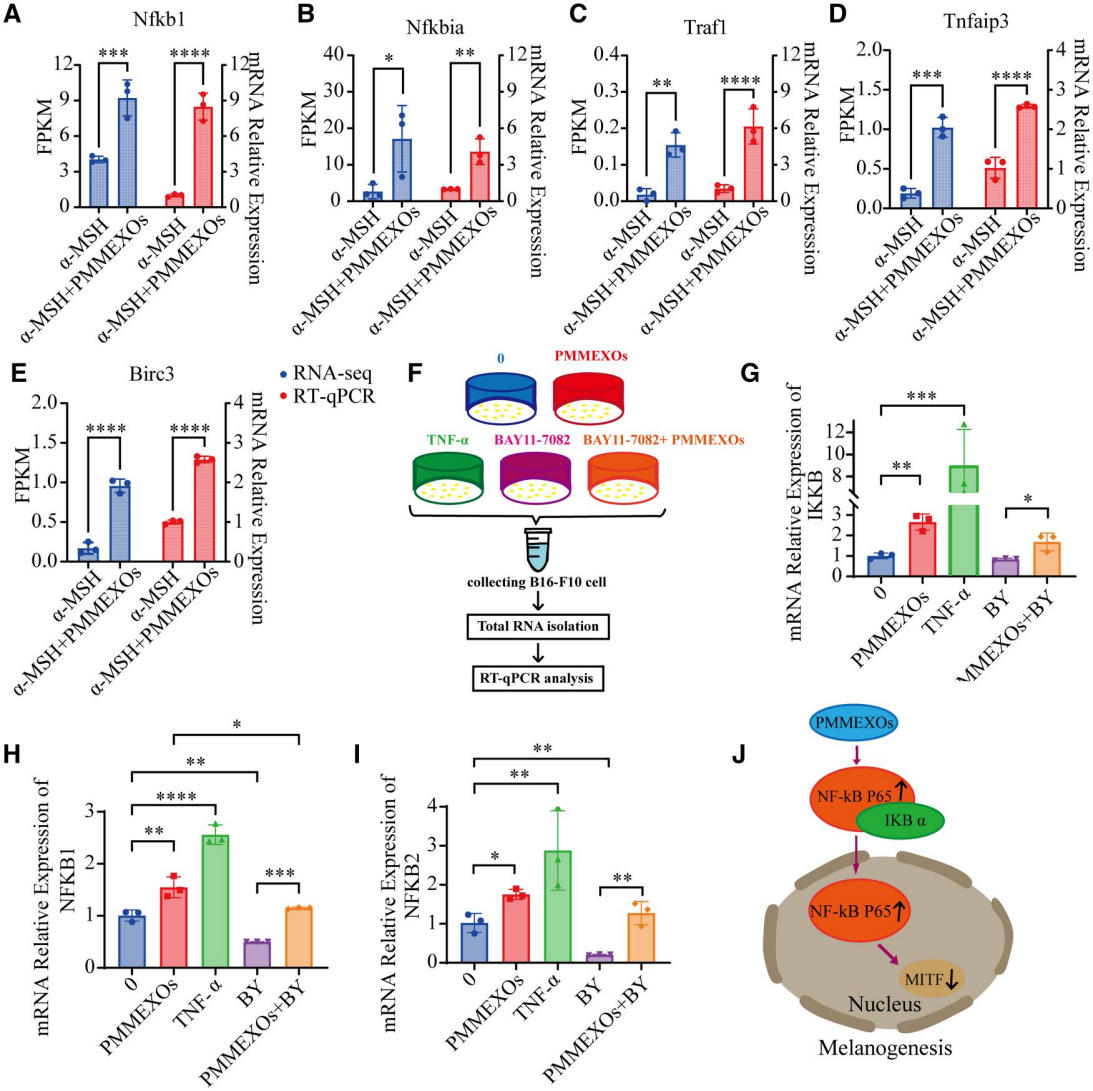
RT-qPCR validated transcriptome sequencing data. Confirmation of transcriptome sequencing data by RT-PCR, including Nfkb1 (**A**), Nfkbia (**B**), Traf1 (**C**), Tnfaip3 (**D**), Birc3 (**E**). (**F**) A brief diagram of RT-PCR experiments performed on B16-F10 cells treated with different groups. mRNA levels of IKKb (**G**), NFkB1 (**H**) and NFkB2 (**I**) in the NF-κB signaling pathway were detected by RT-PCR. (**J**) Schematic diagram of PMMEXOs inhibiting melanin production. *n* = 3, data are expressed as mean ± SD, one-way analysis of variance was used for statistical significance. *P < 0.05, **P < 0. 01, ***P < 0.001, ****P < 0.0001.

To validate the transcriptome data, we randomly selected five DEGs—Nfkb1, Nfkbia, Traf1, Tnfaip3 and Birc3—and RT-qPCR. The expression patterns obtained from RT-qPCR were consistent with the RNA-seq data (Figure 8A–E), confirming the reliability of the transcriptome sequencing results. Given this consistency, we proceeded to explore whether PMMEXOs influence melanin production via the NF-κB pathway after α-MSH induction (Figure 8F). To investigate NF-κB pathway activation, we measured the mRNA levels of NFkB1, NFKB2 and IKKb using RT-PCR. Our results showed that PMMEXOs significantly activated the NF-κB pathway, increasing the mRNA levels of IKKb (Figure 8G), NFkB1 (Figure 8H) and NFKB2 (Figure 8I), similar to the effects of the NF-κB activator TNF-α. Additionally, when the NF-κB pathway was blocked with the inhibitor BAY11-7082, we observed a reduction in the mRNA levels of NFkB1, NFKB2 and IKKb, confirming that PMMEXOs activated the NF-κB pathway after α-MSH induction (Figure 8J), thereby inhibiting melanin production.

### PMMEXOs inhibit melanin production by activating the NF-κB signaling pathway

**Figure 9.**
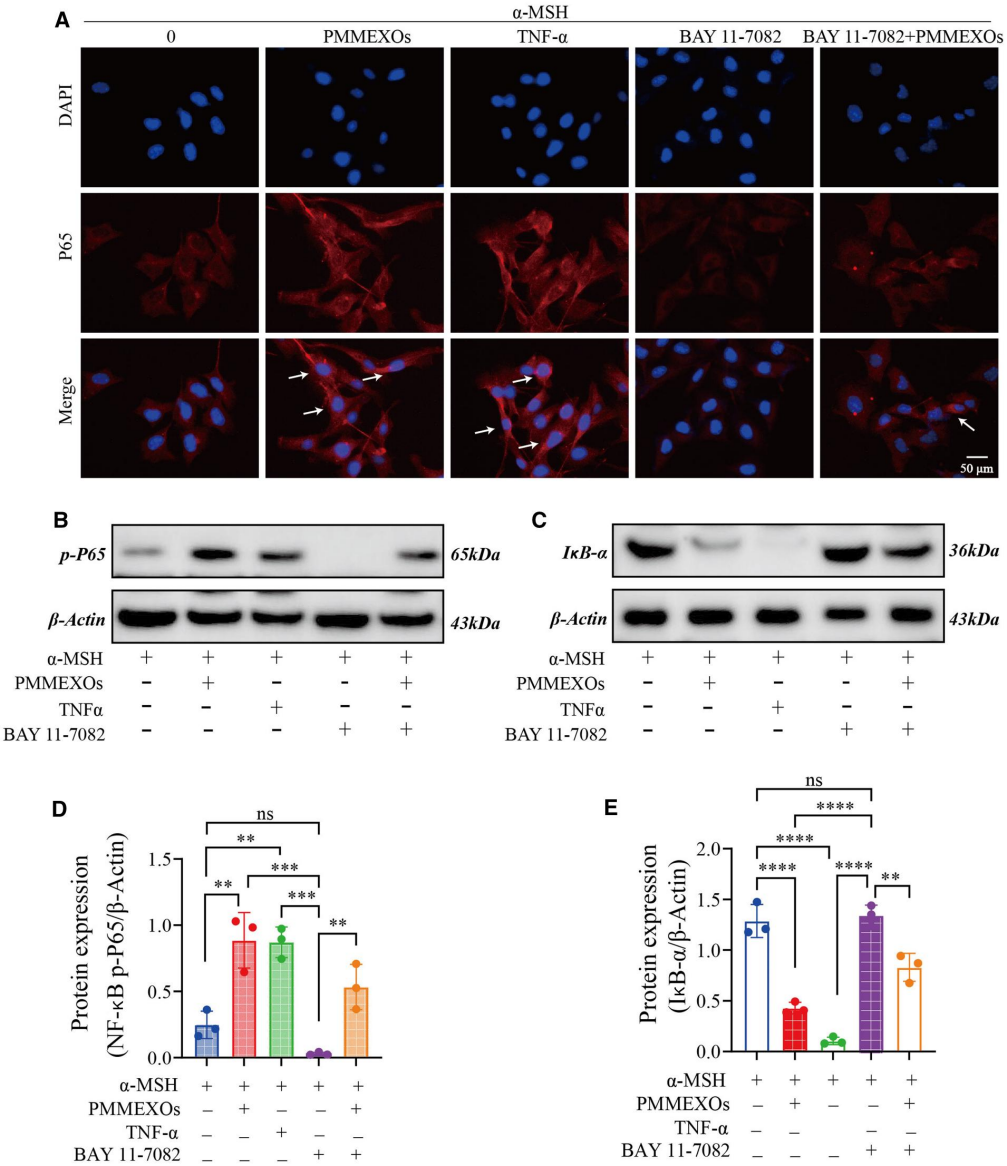
PMMEXOs reduces melanin production by activating the NF-κB signaling pathway. (**A**) The translocation of NF-κB p-65 from cytoplasm to nucleus of B16-F10 cells after different drug stimulation was detected by immunofluorescence assay. The arrow indicates the translocation of NF-κB p-65 from cytoplasm to nucleus. Fluorescent of NF-κB p-65 proteins, nuclei stained with DAPI. Scale bars = 50 μm. WB detection of NF-κB p-P65 (**B**) and IκB-α (**C**) protein expression. The relative protein expression level of NF-κB p-P65 (**D**) and IκB-α (**E**) were quantitatively analyzed. *n* = 3. Data are expressed as mean ± SD, one-way analysis of variance was used for statistical significance. n. s. no significant difference observed between groups. *P < 0.05, **P < 0. 01, ***P < 0.001, ****P < 0.0001.

PMMEXOs treatment facilitates the translocation of p65 to the cell nucleus. To further confirm that PMMEXOs inhibit melanin production through NF-κB pathway activation, we performed cellular IF and WB analyses to assess the expression levels of p65, phospho-p65 (p-P65) and IκBα. We assessed the nuclear translocation of the protein p65 through cellular IF experiments, providing evidence that PMMEXOs inhibit melanin synthesis by activating the NF-κB pathway. In the control group, the NF-κB p65 subunit was predominantly localized in the cytoplasm, with a relatively weak fluorescence signal in the nucleus. In the PMMEXOs-treated group, the fluorescence signal of the p65 subunit was significantly stronger in the nucleus, indicating that the p65 subunit underwent nuclear translocation following PMMEXOs treatment (Figure 9A), which is consistent with existing research findings [54]. WB analysis demonstrated that PMMEXOs treatment increased the expression of p-P65, while decreasing IκBα expression, aligning with the response induced by TNF-α. In contrast, BAY11-7082 suppressed the phosphorylation of p65 and activated IκBα expression, but PMMEXOs treatment reversed these effects (Figure 9B–E). Collectively, these findings suggest that PMMEXOs activate the NF-κB signaling pathway, inhibiting α-MSH-induced melanin deposition, consistent with the results observed in Figure 8.

### miRNAs in PMMEXOs regulate melanin production

**Figure 10.**
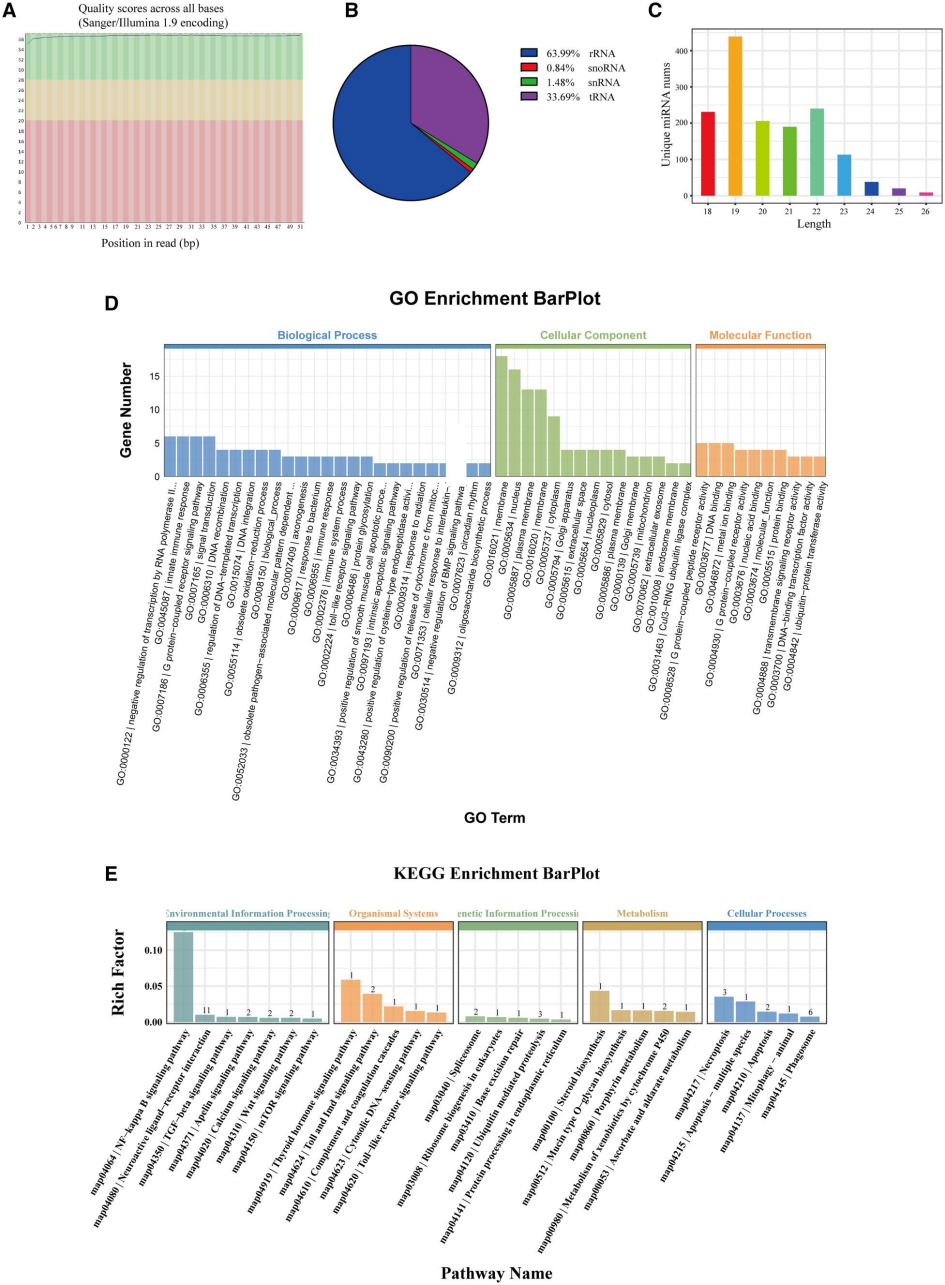
miRNA-Seq analysis of PMMEXOs. (**A**) Quality analysis results of RNA extracted from PMMEXOs. (**B**) All mapped clean reads are annotated and the percentage is calculated. (**C**) Length distribution of identified miRNAs. The GO (**D**) and KEGG (**E**) enrichment barplot of the predicted target genes for the highly expressed miRNAs in PMMEXOs.

Emerging evidence highlights the role of miRNAs in regulating melanin production and skin pigmentation disorders. By deepening our understanding of miRNA activity in gene regulation and melanin synthesis, we can uncover novel miRNAs that influence the signaling pathways of melanin production [55, 56]. To explore the miRNAs enriched in PMMEXOs that may regulate melanin deposition, we conducted miRNA sequencing (miRNA-Seq) to profile the miRNA content in PMMEXOs. As shown in Figure 10A, the Q20 and Q30 quality percentages of the sequencing data were high, indicating excellent sequencing quality. After processing, we obtained a total of 20 207 156 raw reads, with 13 042 641 (64.54%) clean reads and 6 741 027 (33.36%) valid reads. Filtering out non-miRNA sequences, we found that 63.99% of the reads were rRNA, 33.69% were tRNA, 1.48% were snRNA and 0.84% were snoRNA (Figure 10B). The length distribution of the identified miRNAs (Figure 10C) showed a predominant length of 19 nucleotides, which is ideal for binding mRNA targets and influencing gene regulation in biological processes. miRNA expression levels were analyzed using ACGT101-miR (v4.2), identifying 54 highly expressed miRNAs. We then used TargetScan (v5.0) and miRanda (v3.3a) to predict the target genes of the top 10 highly expressed miRNAs.

Further GO and KEGG analyses were conducted to investigate the potential biological functions of these miRNAs. The GO analysis (Figure 10D) indicated that these miRNAs were involved in biological processes, signal transduction and cellular components such as the cytoplasm, membrane composition, extracellular space and extracellular vesicles (GO: 1903561). In molecular functions, they were primarily associated with protein, enzyme and nucleic acid binding. The KEGG pathway enrichment (Figure 10E) revealed that these miRNAs play a role in several signaling pathways, including NF-κB, TGF-β, Wnt, mTOR and Toll-like receptor pathways. Specifically, we used miRanda to predict the potential binding sites on the 3’UTR of NF-κB mRNA. Supplementary Figure S8 shows the predicted binding site sequence and its position on the UTR of the target gene. The results show that the predicted score is 86 points and the energy is -21.129999 kcal/mol. This result effectively proved that miR-96 might bind to a specific region of NF-κB mRNA through seed sequences, thereby providing key bioinformatics evidence for the molecular mechanism by which miR-96 regulates the NF-κB signaling pathway, which is highly consistent with the hypothesis we proposed in the article. These results suggest that the miRNAs in PMMEXOs contribute to the regulation of melanin production.

## Discussion

The dysregulation of α-MSH expression or its signaling pathway can result in various pigmentation disorders, including melasma, freckles and other pigmentary skin diseases [57]. As such, there is an ongoing demand for safe and effective treatments to inhibit melanin production, particularly those that target key enzymes like tyrosinase. Hydroquinone inhibits tyrosinase activity during melanin formation and enhances pigmentation by interfering with the oxidation process of tyrosine in the early stages of melanin biosynthesis. However, hydroquinone is associated with skin irritation and contact dermatitis, and long-term use has been linked to various side effects, such as the development of chloasma. As a result, hydroquinone is prohibited from being used as a raw material in whitening products, with its use restricted to prescription drugs in certain applications [58–60]. Similarly, kojic acid inhibits tyrosinase activity in melanin synthesis, thereby suppressing the final stage of melanin production. However, kojic acid exhibits low skin permeability, limited stability and minimal whitening effects. Furthermore, prolonged use of kojic acid can lead to cytotoxicity, dermatitis and erythema [61, 62]. Recent clinical trends have highlighted the potential of natural products that can suppress melanin synthesis while minimizing side effects [8, 63]. EXOs, nanosized vesicles derived from cells, have arisen as hopeful candidates for therapeutic applications because of their capacity to carry diverse bioactive molecules that can be efficiently delivered to recipient cells. EXOs from human adipose tissue-derived mesenchymal stem cells (hAT-MSCs) have been shown to inhibit melanin production, although their clinical use is often limited by concerns over pathogen transmission from mammalian cell sources [64]. In contrast, EXOs derived from non-mammalian organisms, such as the marine mollusk PM, offer an exciting alternative. These EXOs have recently been shown to inhibit melanin synthesis, but much remains to be understood about their mechanisms of action. Our study offers fresh evidence that PMMEXOs inhibit α-MSH-induced melanin synthesis and tyrosinase activity *in vitro* and *in vivo*, thereby offering novel understandings toward potential therapeutic strategies for pigmentary disorders.

The molecular regulation of melanin synthesis is primarily mediated by α-MSH through its activation of the MITF, a central controller of melanogenesis. MITF, in turn, induces the manifestation of key enzymes such as TYR, TRP-1 and TRP-2, which are critical for the hydroxylation and oxidation of tyrosine and DOPA into melanin. Tyrosinase activity is a rate-constraining step during melanin biogenesis, and the regulation of these enzymes is crucial for controlling pigmentation [65]. Our findings demonstrate that PMMEXOs significantly reduce melanin content and tyrosinase activity (Figure 3D–G), consistent with a downregulation of MITF and its downstream targets. Specifically, treatment with PMMEXOs in B16-F10 melanoma cells led to decreased expression of MITF, TYR, TRP-1 and TRP-2, suggesting that PMMEXOs suppress melanin synthesis through the suppression of MITF-mediated transcriptional activation of melanin biosynthetic enzymes (Figure 4). Notably, prior studies have demonstrated the inhibitory effects of various bioactive compounds on melanin synthesis in zebrafish embryos, including sesamol, which has been shown to suppress pigmentation in a dose-dependent manner, and Phlorofucofuroeckol-A (PFF-A) from Ecklonia cava [66, 67]. To extend our investigation beyond cell culture models, we employed the zebrafish (Danio rerio) as an *in vivo* model for melanin production. Zebrafish embryos share significant genetic homology with mammals in the context of pigmentation genes, including the TYR gene, making them an ideal model for studying melanin synthesis [68]. *In vivo* experiments have confirmed that PMMEXOs, a novel type of naturally derived EXOs, not only inhibit melanin synthesis and tyrosinase activity in zebrafish embryos at low concentrations but also in a density-reliant way (Figure 6). Additionally, PMMEXOs has demonstrated strong depigmentation effects in mouse pigmentation models, which is consistent with the results observed in B16-F10 cells. This suggests that PMMEXOs could serve as potential agents for inhibiting melanin synthesis.

MITF directly regulates melanin production by activating key genes involved in melanin synthesis, such as tyrosinase (TYR), and its expression is controlled by multiple signaling pathways [69]. For example, JNK and p38 mitogen-activated protein kinase (MAPK) are involved in activating MITF expression, which subsequently increases tyrosinase expression [70]. The ERK activation signal also enhances CREB phosphorylation, leading to an increase in MITF expression [71]. Both NF-κB and MAPK (ERK/JNK/p38) pathways jointly regulate the expression of pro-inflammatory cytokines, such as TNF-α and IL-6, which can feedback and further promote melanin production in the inflammatory microenvironment [72]. UVB activates the Akt/IKKα/NF-κB axis through oxidative stress while simultaneously activating the MAPK pathway, creating a synergistic effect. Research has shown that ODN2006 (a TLR9 agonist) enhances the activity of NF-κB p65 and melanin synthesis in conjunction with UVB exposure [73]. Additionally, cAMP can induce NF-κB binding to the κ light chain enhancer, activating the κB reporter gene. Studies indicate that the tyrosinase inhibitor MHY884 significantly reduces UVB-induced melanin production by inhibiting Akt/IKKα phosphorylation and blocking NF-κB activation [74]. Therefore, interactions between NF-κB and other signaling pathways, such as cAMP/PKA/CREB and MAPK, may play a crucial role in regulating melanin production. To further elucidate the mechanism by which PMMEXOs regulate melanin production, we conducted mRNA sequencing and performed KEGG enrichment analysis. The results indicated that the primary differential pathway is the NF-κB signaling pathway. The NF-κB signaling pathway is thoroughly confirmed as a key modulator of immune responses and inflammation [75]. More recently, this pathway has been implicated in the regulation of melanogenesis. It has been shown that activation of the NF-κB pathway by inflammatory mediators, such as IL-18 and toll-like receptor (TLR) agonists, enhances melanogenesis [73]. Conversely, TNF-α-induced hypopigmentation is dependent on the suppression of NF-κB signaling [38], indicating that NF-κB can have both activating and inhibitory effects on melanin synthesis depending on the context. Our data align with these findings, as we observed that α-MSH induction led to an increase in melanin production in B16-F10 cells, accompanied by a reduction in the expression of key NF-κB signaling genes, including IKKβ, NFkB1 and NFkB2. This proposed that NF-κB signaling may be suppressed under α-MSH conditions. Importantly, treatment with PMMEXOs resulted in the activation of the NF-κB pathway, as evidenced by RNA sequencing, RT-PCR, IF and WB analyses (Figures 8 and 9) indicate that PMMEXOs activate NF-κB signaling, thereby regulating the expression of MITF and its downstream melanin biosynthetic enzymes (Figure 8I). The involvement of NF-κB signaling in the inhibition of α-MSH-induced melanin deposition provides a novel mechanistic insight into the action of PMMEXOs, indicating that the activation of NF-κB p65 by PMMEXOs may be crucial for their anti-pigmentation effects.

EXOs are rich in noncoding RNAs, particularly miRNAs, which are key controllers of gene expression participating in diverse cellular processes, including melanogenesis. miRNAs have emerged as significant players in the regulation of pigmentation, as they can modulate the expression of melanin biosynthetic enzymes, including MITF, and affect melanocyte function [76]. The ability to easily detect miRNAs in bodily fluids has further fueled their potential as biomarkers and therapeutic agents [77]. MiRNA regulates keratinocyte function and enhances wound healing by inhibiting its targeting of SIRT1, highlighting the critical role of miRNA in cellular function modulation and wound repair [32]. In this study, miRNA sequencing of PMMEXOs revealed a robust enrichment of miRNAs involved in the regulation of several critical signaling pathways, including NF-κB, TGF-β, Wnt, mTOR and TLR pathways (Figure 9E). These miRNAs may collectively regulate melanin synthesis through complex molecular networks. It is widely recognized that miRNA modulates the stability of mRNA by attaching to the 3’-UTR region of its targeted mRNA [78]. NF-κB, a pivotal signaling molecule, synergizes with TLR4 to modulate the inflammatory process and promote wound healing [79]. The underlying mechanism is not known and remains to be elucidated. Interestingly, we found that the miRNAs within PMMEXOs are enriched in pathways known to influence melanogenesis. For example, TLR signaling, particularly via TLR9, has been shown to enhance melanin production through NF-κB activation [80]. Furthermore, NF-κB translocation to the nucleus can alter the expression of target genes that control melanocyte activity, including melanotropins that influence pigmentation [81]. Our findings suggest that PMMEXOs may exert their anti-pigmentation effects through a combination of NF-κB activation and miRNA-mediated regulation of melanin biosynthesis.

This study provides compelling evidence that PMMEXOs inhibit melanin production through a combination of NF-κB signaling activation and miRNA-mediated regulation. By elucidating the molecule-based processes beneath the anti-pigmentation effects of PMMEXOs, we highlight their potential as a novel curative tactic for the remedy of pigmentation disorders. However, while our findings are promising, additional research is required to completely understand the precise mechanisms by which miRNAs within PMMEXOs modulate melanin synthesis. Future studies should focus on identifying the specific miRNAs involved, their target genes, and the broader molecular networks they regulate. This will enable the development of targeted, effective therapies for skin pigmentation disorders.

## Conclusion

This study successfully isolated and characterized PMMEXOs and demonstrated their effective internalization into B16-F10 cells. Our research indicates that PMMEXOs regulate melanin production by activating the NF-κB signaling pathway, which in turn modulates MITF, leading to a significant anti-melanin effect. Marine-derived EXOs have shown potential in treating skin diseases, yet the components and mechanisms by which they inhibit melanin production remain unclear. In conclusion, this suggests that PMMEXOs have new clinical application potential in the precaution and remedy of skin diseases, including wrinkles, melasma and age spots.

## Supplementary Material

rbaf072_Supplementary_Data

## Data Availability

The raw data and processed data required to reproduce these findings are available from the corresponding author upon request.
