# Supplementary material for: Biocompatible exosomes derived from Pinctada martensii mucus for therapeutic melanin regulation via α-MSH/NF-κB/MITF pathway
Source: Regen Biomater. 2025 Jul 3;12:rbaf072. doi: 10.1093/rb/rbaf072 (PMC12311293; doi:10.1093/rb/rbaf072)
Supplement: rbaf072_Supplementary_Data [file rbaf072_supplementary_data.docx]

**Biocompatible Exosomes Derived from Pinctada Martensii Mucus for Therapeutic Melanin Regulation via α-MSH/NF-κB/MITF pathway.**

Dandan Mo ^1, 2 #^, Weihao Zheng^1, 2 #^, Zixin Gao ^3 #^, Ke Ma^1, 2, 4^, Ke Yang ^4^, Tao Zeng^1^, Chaozheng Qin^1, 3^, Yan Luo ^1^ *, Li Zheng ^1, 2, 3^ *, Sheng Xu ^1, 3^ *

^1^ Collaborative Innovation Centre of Regenerative Medicine and Medical Bioresource Development and Application Co-constructed by the Province and Ministry, Guangxi Key Laboratory of Regenerative Medicine, Guangxi Engineering Center in Biomedical Materials for Tissue and Organ Regeneration, The First Affiliated Hospital of Guangxi Medical University, Nanning, 530021, China.

^2^ Pharmaceutical College, Guangxi Medical University, Nanning, 530021. China.

^3^ Life Sciences Institute, Guangxi Medical University, Nanning, 530021, China.

^4^ Department of Plastic & Cosmetic Surgery, The First Affliated Hospital of Guangxi Medical University, Nanning, 530021.China.

* Corresponding authors.

Email addresses: xusheng@gxmu.edu.cn (S. Xu), [luoyan2007@163.com](mailto:luoyan2007@163.com) (Y. Luo), and zhengli224@163.com (L. Zheng)

^#^ The authors contributed equally to this work.

| 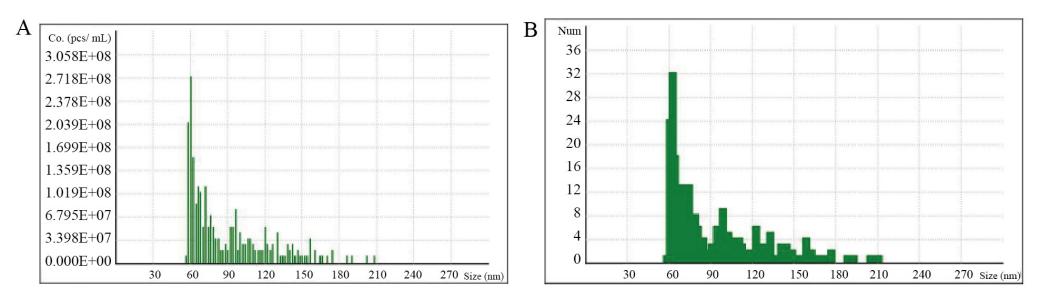 |
| --- |
| **Figure S1.** Measurement of PMMEXOs concentration (A) and particle size analysis (B) by Nanocoulter I particle size analyzer. |

| 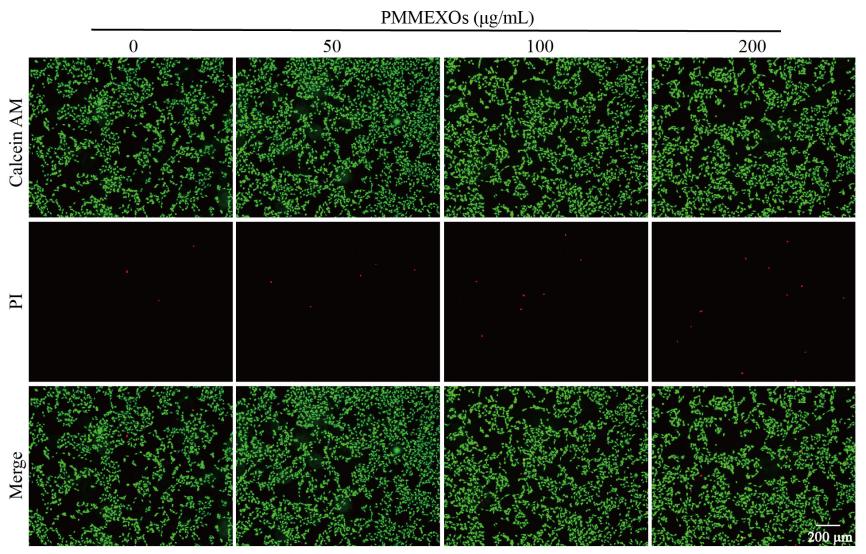 |
| --- |
| **Figure S2.** B16-F10 cells were treated with different concentrations of PMMEXOs for 24 hours, and cell viability was determined by live/dead assay. Green, living cells; Red, dead cells. Scale bars =200 μm. |

| 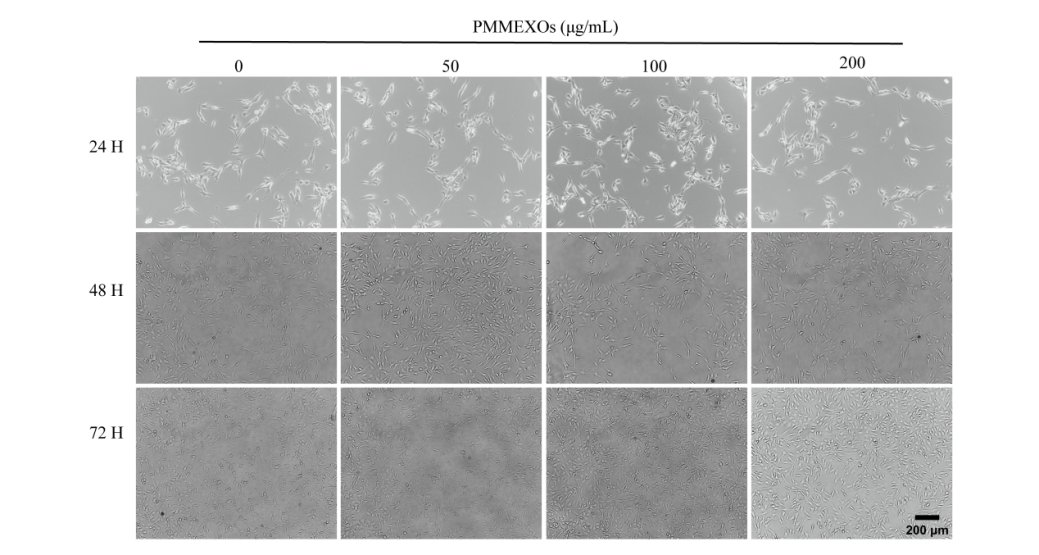 |
| --- |
| **Figure S3.** α-Arbutin (0, 50, 150, 500, 1000, 2000 μM) treated B16-F10 cells at 72 h relative cell viability. n=6. |

| **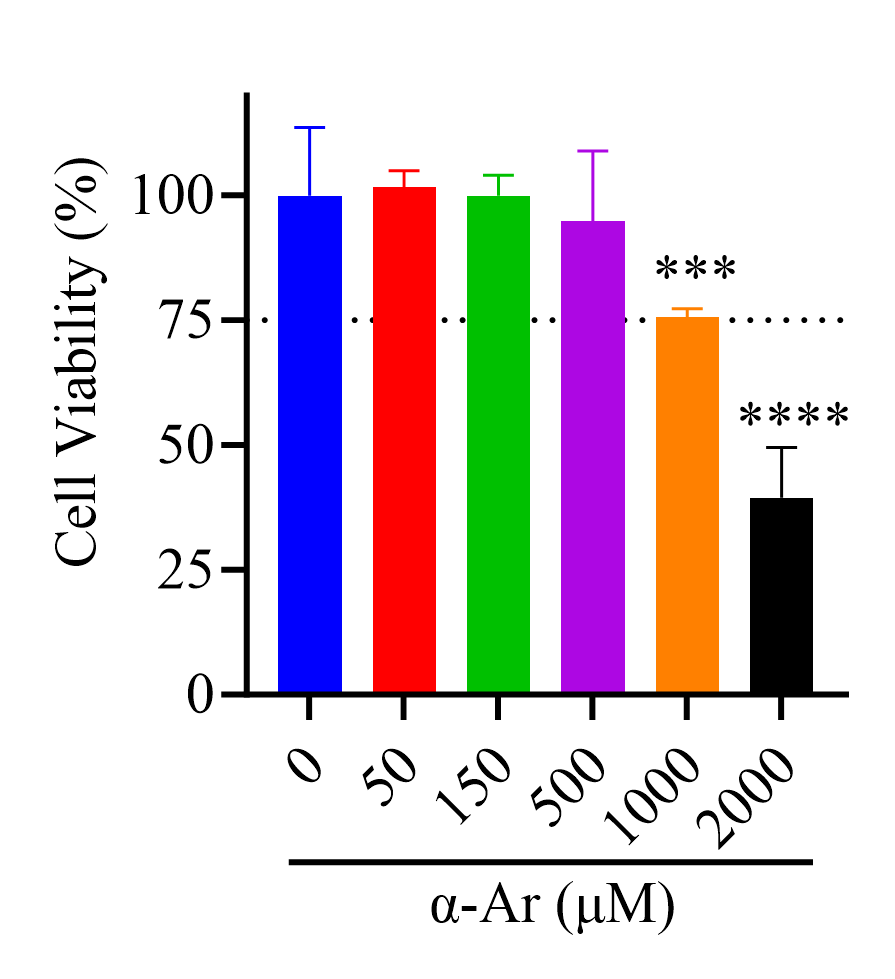** |
| --- |
| **Figure S4.** α-Arbutin (0, 50, 150, 500, 1000, 2000 μM) treated B16-F10 cells at 72 h relative cell viability. n=6. |

| 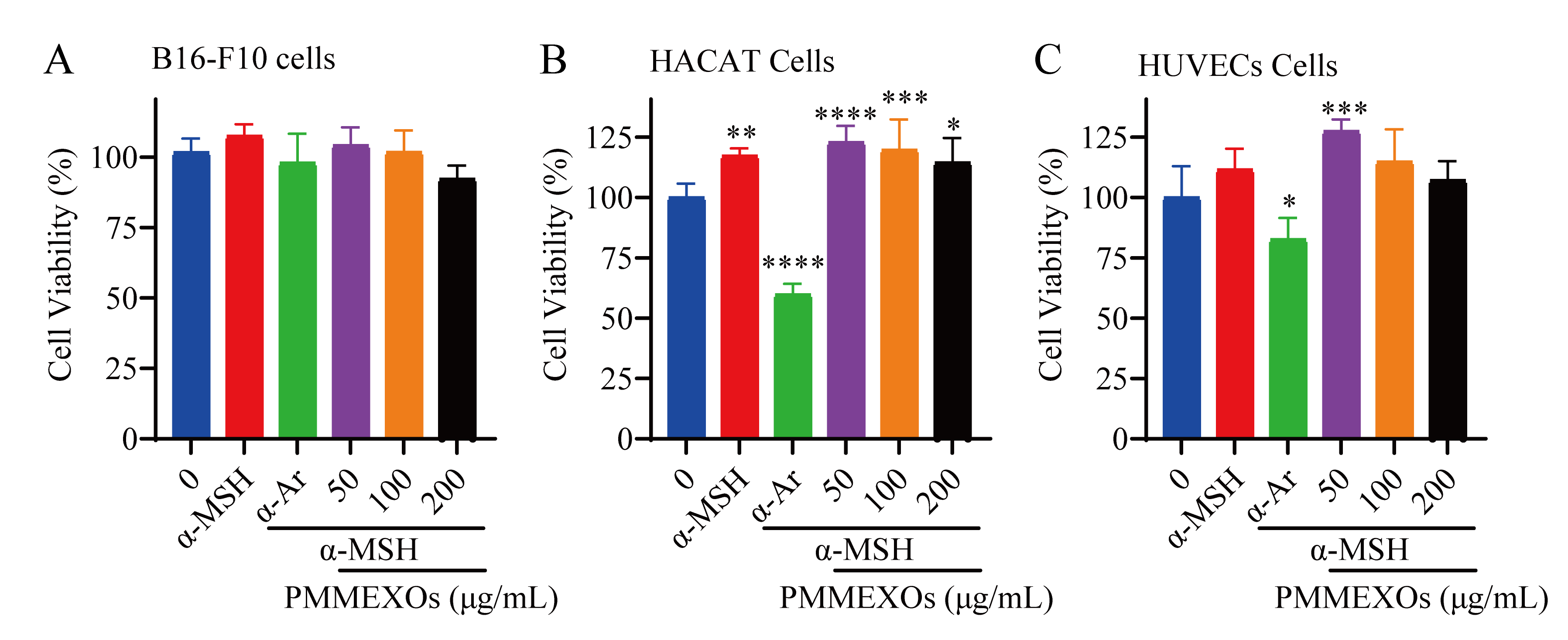 |
| --- |
| **Figure S5.** Cellular activity of B16-F10 cells (A), HACAT cells (B) and HUVECs cells (C) in each treatment group. n=6. |

| 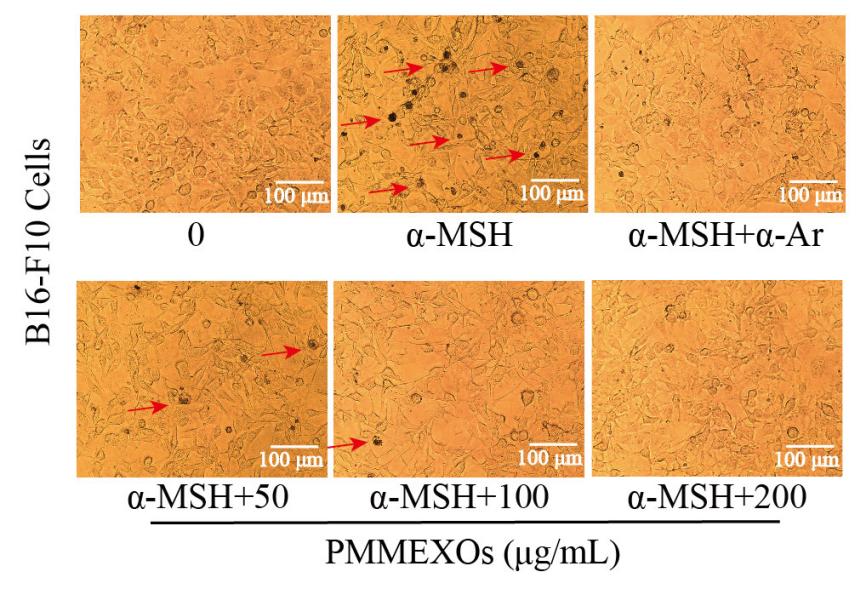 |
| --- |
| **Figure S6.** Representative image of intracellular melanin granules on B16-F10 cells. Scale bars =100 μm. |

| 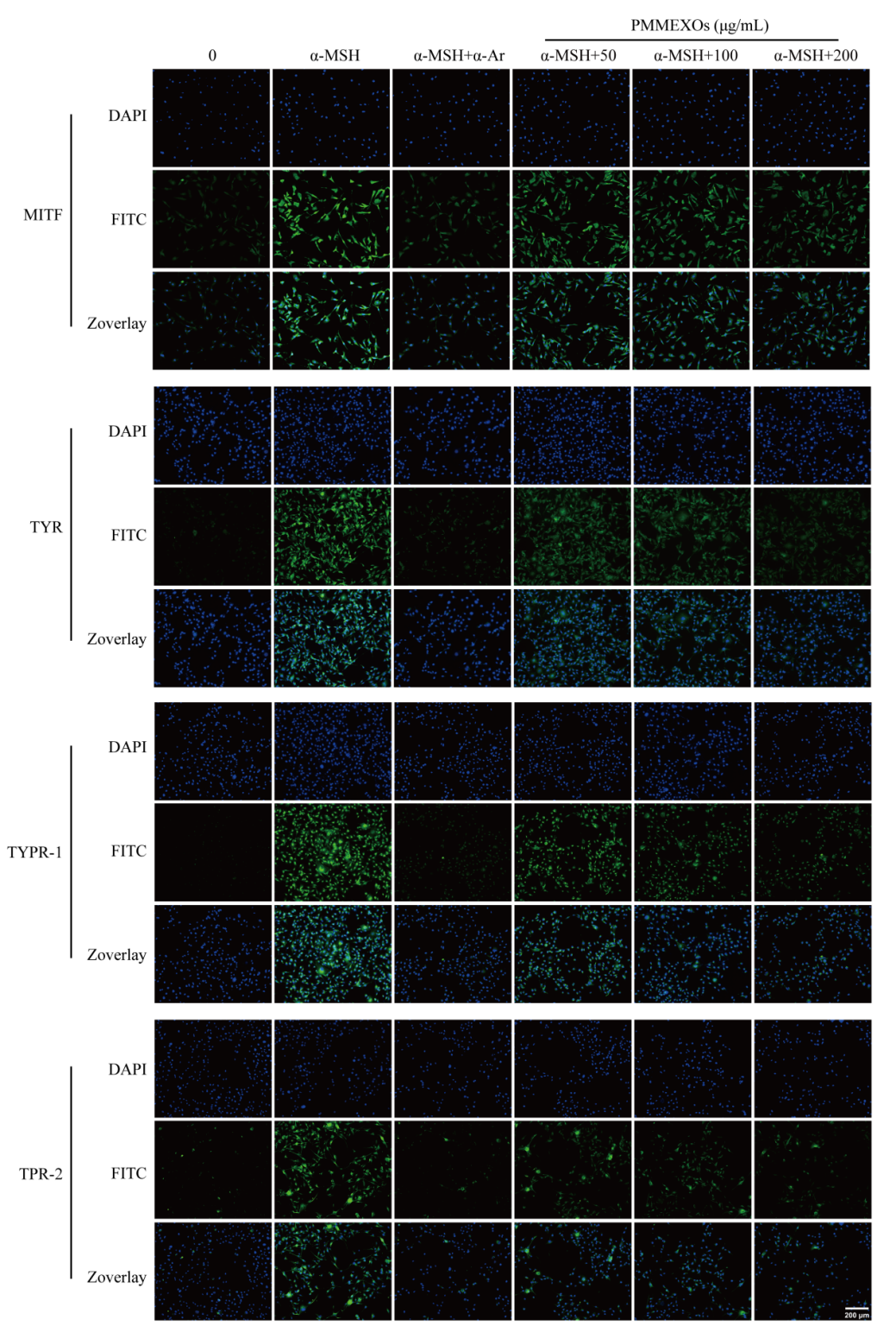 |
| --- |
| **Figure S7.** Immunofluorescence staining of MITF, TYR, TYRP-1 and TRP-2 proteins during melanin production. After incubation for 24 h, fluorescent of MITF, TYR, TYRP-1 and TRP-2 proteins (green), nuclei stained with DAPI (blue). Scale bars = 200 μm. n=3. |

| **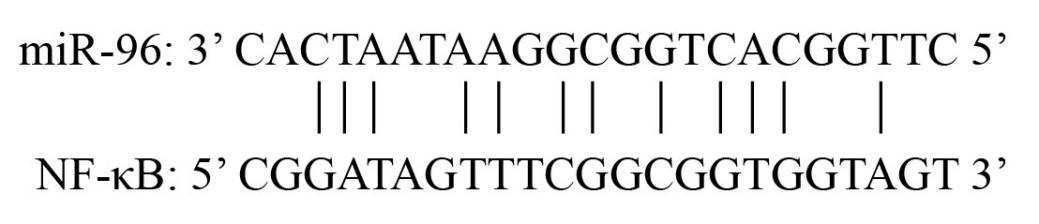** |
| --- |
| **Figure S8.** Prediction map of the binding sites of miR-96 and NF-κB. |

**Table S1.** Premers in qRT-PCR analysis.

| GENE | Primer sequence |
| --- | --- |
| GAPDH-F | AGGTCGGTGTGAACGGATTTG |
| GAPDH-R | TGTAGACCATGTAGTTGAGGTCA |
| MITF-F | ACTTTCCCTTATCCCATCCACC |
| MITF-R | TGAGATCCAGAGTTGTCGTACA |
| TYR-F | CTCTGGGCTTAGCAGTAGGC |
| TYR-R | GCAAGCTGTGGTAGTCGTCT |
| TYRP-1-F | CCCCTAGCCTATATCTCCCTTTT |
| TYRP-1-R | TACCATCGTGGGGATAATGGC |
| TRP-2-F | TTCTGCTGGGTTGTCTGGG |
| TRP-2-R | CACAGATGTTGGTTGCCTCG |
| Ikbκb-F | ACAGCCAGGAGATGGTACG |
| Ikbκb-R | CAGGGTGACTGAGTCGAGAC |
| Nfκb1-F | ATGGCAGACGATGATCCCTAC |
| Nfκb1-R | TGTTGACAGTGGTATTTCTGGTG |
| Nfκb2-F | GGCCGGAAGACCTATCCTACT |
| Nfκb2-R | CTACAGACACAGCGCACACT |
| Tnfaip3-F | ACAGTGGACCTGGTAAGAAAACA |
| Tnfaip3-R | CCTCCGTGACTGATGACAAGAT |
| Birc3-F | ACGCAGCAATCGTGCATTTTG |
| Birc3-R | CCTATAACGAGGTCACTGACGG |
| Nfκbia-F | GGAGGCATGTTCGGTAGTGG |
| Nfκbia-R | TTCGTGGATGATTGCCAAGTG |
| Traf1-F | AGGGTGGTGGAATTACAGCAA |
| Traf1-R | GCAGTGTAGAAAGCTGGAGAG |
